# Scots pine seedlings of lowland and upland ecotypes respond differently to drought detected by needle functional traits and spectral reflectance

**DOI:** 10.1186/s12870-025-08019-y

**Published:** 2026-01-14

**Authors:** Kristýna Štěpánová, Zuzana Lhotáková, Eva Neuwirthová, Lucie Kupková, Lucie Červená, Filip Raasch, Markéta Potůčková, Jan Stejskal, Jaroslav Čepl, Petya Campbell, Milan Lstibůrek, Jana Albrechtová

**Affiliations:** 1 Department of Experimental Plant Biology, Faculty of Science, Charles University, Viničná 5, Prague, 12844 Czech Republic; 2 Department of Forest Genetics and Physiology, Faculty of Forestry and Wood Sciences, Czech University of Life Sciences Prague, Prague, Czech Republic; 3 Department of Applied Geoinformatics and Cartography, Faculty of Science, Charles University, Albertov 6, Prague, 12800 Czech Republic; 4 Department of Geography and Environmental Sciences, University of Maryland Baltimore County and NASA/Goddard Space Flight Center, Biospheric Sciences Laboratory, Greenbelt, MD 20771 USA

**Keywords:** Seedling screening, Hyperspectral data, *Pinus sylvestris* L., Chlorophyll fluorescence, Needle anatomy, Phenotyping, PRI, REP, Ecotypes, Drought resistance

## Abstract

Scots pine (*Pinus sylvestris* L.) is widely distributed, phenotypically plastic forest tree species with modest ecological demands, therefore it is a very suitable, drought tolerant species for afforestation at present. This is especially important given Europe’s changing climate, with rising extremes and unpredictable rainfall challenging forest regeneration. Drought resistance of seedlings is essential for their survival during current reforestation efforts, however, its relation to ecotypic variation is yet not well understood. The objective of this study was to investigate the response of seedlings from two Czech Scots pine ecotypes (upland and lowland), exposed to water deficit at the beginning of the vegetative season – a critical period for successful afforestation from the perspective of precipitation availability. During a greenhouse experiment with nursery pre-grown seedlings, terminal shoot length and selected leaf functional traits (leaf mass per area; water and pigment contents; needle anatomy), chlorophyll fluorescence kinetics and seedling reflectance were monitored during ten-week irrigation reduction and after rewatering. The photochemical reflectance index (PRI) and the red edge position (REP) were calculated from spectral reflectance to distinguish differently treated seedlings. The lowland ecotype grew faster under control but suffered stronger growth reduction and higher mortality under drought. In contrast, across all recorded responses, the upland ecotype responds more consistently to changes in water availability, does not reduce terminal growth, accumulates less biomass and exhibits lower mortality. In general, for terminal growth, there was a significant effect of treatment and also an interaction of treatment and ecotype during the recovery period, unlike the drought period. REP was responsive in recovery period for upland ecotype while PRI showed no consistent drought-related pattern. Our results, in agreement with the fluorescence-based indicators, suggest that current-year needles are more suitable for drought stress detection using spectral indices. The upland ecotype showed several functional traits corresponding to better resilience to drought stress compared to the lowland ecotype. Understanding drought stress and recovery responses via effective leaf functional traits will help forest management to select suitable ecotypes for reforestation, ensuring a higher survival under changing climatic conditions.

**Supplementary Information:**

The online version contains supplementary material available at 10.1186/s12870-025-08019-y.

## Background

Global climate change is severely affecting temperate forest ecosystems due to warming, accompanied by alterations in precipitation patterns, increasing frequency and intensity of drought and heatwaves, which are expected to expand drought-affected areas in the coming decades [1]. Forest species chosen for reforestation must be able to adapt to these multifactorial stresses to survive [2]. In Europe, heatwaves and droughts have intensified, with projections indicating a significantly higher probability of extreme heat events by 2040 [3]. Europe is warming three to four times faster than other northern mid-latitude regions [4]. This rapid warming has been clearly illustrated by the heatwaves of 2022, 2023, and 2024 [5–7], which caused severe ecological and societal impacts, including widespread wildfires even in protected areas [8]. These events highlight the increasing risk of climate change-induced droughts and disruption of forest ecosystems [9] involving drought-induced tree mortality, which increased in Scots pine also over the past two decades [10, 11].

Plant species are predicted to shift their geographic range as a result of climate change impacts [12]. In Europe, many high-latitude species are projected to experience range contractions, while lower-latitude species expansion; for Scots pine (*Pinus sylvestris* L.), models indicate a northward shift with increasing loss risk in the southern and eastern range, ranking among the most affected species together with Norway spruce (*Picea abies* L. Karst) [13]. However, the rate of natural migration of resilient tree species or their ecotypes is not as fast as the climatic change progress [14]. Due to climate change, the risk of maladaptation in European forest trees is increasing [15]. Assisted migration, involving the use of more resistant tree genotypes [16], is recommended as a reliable management strategy for afforestation and reforestation processes in Central Europe, which could lead to preserving forest carbon sink [17]. These models, however, strongly rely on the quality and accessibility of provenance trials. Scots pine was one of the first species investigated in terms of provenance clinal variation even before the famous IUFRO series [18], but only relatively recent provenance studies have incorporated physiological traits [19]. The success of reforestation relies on the use of suitable seedlings with high morpho-physiological quality in forest nurseries [20].

After the recent Norway spruce (*Picea abies* L. Karst) bark beetle-induced dieback [21, 22], Scots pine became one of the most used tree species for afforestation and reforestation in Central Europe [23] though it can also be attacked by bark beetle (*Ips acuminatus* and *Ips sexdentatus*) [24]. Scots pine has one of the largest distribution ranges and grows naturally across various climatic conditions in various ecotypes [25, 26]. Scots pine is characterised by modest ecological demands on the environment and climate of the habitat, and is considered as a relatively drought resistant with its drought tolerance linked to both its phenotypic plasticity and ecotypic differentiation [27]. Scots pine is considered a typical isohydric plant revealing conservative stomatal behavior [28]. Isohydric behavior is characterized by early drought response in the form of stomatal closure [29], transpiration and photosynthesis rate reduction [30]. Adaptation of the hydraulic system of Scots pine across Europe is to regulate the stomatal conductivity, and it also occurs by adjusted hydraulic architecture (changing the ratio of leaf area to sapwood area) [31]. Intraspecific variability in hydraulic architecture is geographically dependent [11, 29] and genetic plasticity of hydraulic architecture optimization to drought stress in Scots pine across the Europe provenances was described already [30]. An important aspect in ecotypic differentiation of *P. sylvestris* is supposed to be a geographical origin with associated climatic factors shaping specific tree ecotype interactions with fungal and bacterial endophytes, potentially affecting plant health and adaptability across diverse environments [32]. Recent genomic studies have begun to elucidate the genetic basis of local adaptations in Scots pine and have revealed substantial heritable differences in drought response among different provenances [33, 34]. Some genotypes can grow in areas with an annual precipitation ranging between 200–1,780 mm and optimal annual temperature between 5° C and 35° C, however it could survive much larger extremes depending on water availability [35]. In the Czech Republic, Scots pine occupies 16% of the forested area [36], and three ecotypes are identified there: 1) Lowland, a fast-growing pioneer that thrives in monocultures and does not tolerate competition; 2) Wooded-hill, another pioneer variant; and 3) Upland, a climax type that grows in mixtures with other trees at higher altitudes but can also be found at lower elevations [23, 37].

Resilience – the ability to withstand and recover from environmental disturbances – is vital for plant response to changing conditions and forest adaptation to climate change [38, 39]. Recovery rate is central to resilience, influencing both future stress resistance and long-term ecosystem stability [39]. Among conifers, *Pinus* species are known for their flexible water use and ability to compensate for drought-induced declines in net primary production by extending the growing season, provided they can benefit from variable patterns of drought and rainfall [40].

According to Camarero et al. [41], smaller conifers with a higher xylem thickness-to-leaf life span ratio tend to show greater post-drought growth resilience than taller species, likely due to enhanced resistance to xylem cavitation. These findings suggest that structural traits such as plant height and xylem architecture are key determinants of drought resilience and could guide species selection in drought-prone areas. Moreover, the timing of drought events has a greater impact on tree growth and water-use efficiency than drought intensity itself [42, 43]. Drought does not only affect the current year’s growth but can also carry over, negatively impacting the following growing season [44].

Plants respond to drought in many ways, determined by different genetic, biochemical, and physiological adjustments [45, 46]. Understanding the mechanisms of trees´ responses to drought is essential for prediction of drought impact on their growth, reproduction and forest ecosystem functioning [47]. Drought also has a serious impact on the resistance of conifers to various pests, for example, by affecting resin flow in trees [48, 49]. Drought stress can be effectively assessed through leaf functional traits – biochemical, structural, and physiological parameters that serve as indicators of plant responses [50]. In Scots pine, key traits include leaf water potential, contents of photosynthetic pigments, soluble sugars, free amino acids, glutathione [51], proline [52], and chlorophyll fluorescence [53] or needle xylem structure [54]. Leaf functional traits are also used to identify phenotypes with suitable genotypes in breeding programmes aimed on selection of suitable individuals with resistance potential and to cope with extreme site conditions in the long term [55, 56].

Visible to short wave-infrared (Vis-SWIR) reflectance spectroscopy provides a rapid and non-invasive assessment of changes in leaf functional traits at different hierarchical scales from leaf to canopy level [57, 58], allowing detection of early forest stress [59–61] and has increasing potential in human managed forests [62]. Vis-SWIR reflectance was successfully used for determination of Scots pine physiological status on post-mining sites with different pollution load [63], for disentangling the phenotypic and the underlying genetic variation within Scots pine seedlings [55, 64] with possible use for hyperspectral phenotyping [65]. Hyperspectral imaging was used to detect intensity of water stress in Scots pine through monitoring dynamics in photosynthetic pigments such as chlorophylls and carotenoids [66]. The study showed a pattern in linking xanthophyll-related changes in reflectance captured by the photochemical reflectance index (PRI) and chlorophyll changes in red edge position (REP) in response to water stress. Hyperspectral imaging was also efficient for high-throughput screening of loblolly pine (*Pinus taeda* L.) seedlings for freeze tolerance, providing objective assessments of freeze-induced damage and enabling accurate classification of seedling resistance [67]. Another possibility is the use of hyperspectral images from an overhead view for pathogen-induced diseases [68].

Despite frequently studied clinal variation in growth, adaptability, and several physiological traits related to drought response, the ecotypic variation in Scots pine is still understudied and not fully understood. Moreover, there is an urgent need for reliable drought and non-invasive, non-destructive proxies of drought response that could be applied for resilient seedling selection. Our study aimed to evaluate the early-season drought response of Scots pine seedlings originating from two locally adapted ecotypes (i.e., lowland and upland) from the Czech Republic under controlled conditions. We conducted a greenhouse experiment on two-year old seedlings, including ten-week irrigation reduction and followed by rewatering. We tested the following hypotheses: (H1) Lowland and upland ecotypes respond differently to early season drought, which could be detected at the level of leaf functional traits represented by non-specific stress indicators such as pigment content, leaf mass per area, chlorophyll fluorescence kinetics and needle anatomy. (H2) There is a differential ecotype growth response resulting from early season drought stress. We further expect that (H3) water-stress responses in seedlings can be detected non-invasively by reflectance vegetation indices, testing two potential candidates – already well-established in ecophysiological studies: PRI and REP to reflect changes in chlorophyll and carotenoid contents and discriminate differences in responses between different irrigation treatments within the pine ecotypes.

## Methods

### Plant material, cultivation conditions, drought treatment

The pot experiment with nursery pre-grown seedlings of Scots pine originating from two locally adapted ecotypes (lowland and upland) took place in the greenhouse of the Faculty of Science, Charles University in Prague from 14 April to 22 September, 2021. Two contrasting ecotypes were distinguished by Brichta et al. [23] that differ mostly by stand type: the lowland ecotype, typical of pioneer stands on sandy soils with open-canopy conditions and regeneration on exposed mineral substrates, and the upland (montane) ecotype, characteristic of mixed, late-successional forests at higher elevations (700–1,000 m) where natural regeneration occurs under canopy cover in cooler, mesic environments. In our previous studies we provide detailed evidence of a strong population structure based on genomic data, which clearly separated the upland and lowland ecotype [33].

The seeds for nursery grown seedlings originated from different sources: 1) upland ecotype—seed orchard Přimda (848 m a.s.l.; 49.6605600N, 12.6810081E). The seed orchard represents graftings of the superior individuals (plus trees) selected from Scots pine population approximately in a 15 km radius of the seed orchard, based on their growth, vitality, and morphotype typical for upland pine. 2) lowland ecotype – the seed material came from randomly selected plus trees as a bulked sample from the certified stand Písek area (around 500 m a.s.l.; 49.2142269 N, 14.2837978 E). Although the seed sources differ slightly in altitude (~ 300 m), the ecotype differentiation was mainly based on stand type [23] and population structure [33].

**Fig. 1:**
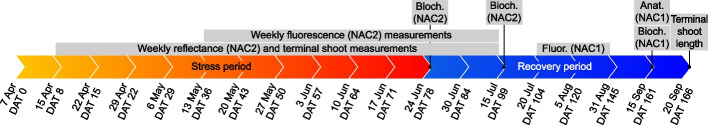
Timeline of the experimental period from 7 April (day after transplantation, DAT 0) to 15 September, 2021 (DAT 167). The drought treatment started on 15 April (DAT 8) and finished on 24 June, 2021 (DAT 78). Then all seedlings were watered to soil saturation and kept under equal irrigation regime, the relative soil water content was continuously measured throughout the time. From 22 April (DAT 15) to 15 July (DAT 99) weekly measurements of terminal bud/shoot length and shoot reflectance was conducted on previous-year needles (Needle-age-class; NAC2). Starting week 5 of the treatment (DAT 43), fluorescence measurements were conducted weekly until 15 July (DAT 99) also on NAC2 needles. At the end of the stress period on 24 June (DAT 78) were sampled needles (NAC2) for evaluating chlorophyll, LMA and water content (Bioch.) and the sampling was repeated after two weeks of recovery on 15th July (DAT 99). The current-year needles (NAC1) were screened for fluorescence (Fluor.) during the recovery phase on 5 and 31 August (DAT 120 and DAT 145) and final sampled for chlorophyll, LMA and water content (Bioch.) and needle anatomy (Anat.) on 15 September (DAT 161) and final measurement of terminal shoot length on 20 September (DAT 166)

Two-year old seedlings were planted in 1L pots, in a substrate of peat, sand, silica sand and perlite in the ratio: 12:2:5:7. The irrigation reduction simulating drought was applied from 15 April to 24 June, 2021. The complete rewatering and equal irrigation of all seedlings (hereafter as recovery) was applied from 25 June to 15 September (Fig. 1). The experiment was a randomized complete block design, with each block including all treatment combinations of the two ecotypes and watering regimes. Altogether 100 seedlings were involved at the beginning of the experiment and for each ecotype and treatment 15 individuals were used for reflectance and needle trait assessments (90 in total). The remaining seedlings were used for soil water content monitoring using WaterScout SM100 Soil Moisture Sensor (described further). These seedlings were excluded from regular measurements to avoid possible artifacts in manipulation pots with moisture sensors.

From the 15 April to the 24 June, three different water regimes were applied: control and two drought levels. The weekly water supply for seedlings was 75–200 ml in the control group (C), 25–100 ml mild drought (MD) stress, and 0–100 ml drought (D) stress conditions. During the stress period each individual tree in the control group received in total 1,675 ml water, while individuals in the mild drought treatment only 950 ml and in the drought treatment 450 ml. From the afternoon of 24 July, after sampling during the stress period, all three groups of seedlings were regularly watered with the same amount of water for recovery after an 11-week stress period.

Substrate moisture was measured using WaterScout SM100 Soil Moisture Sensor capacitive moisture probes (Spectrum Technologies, Inc., USA) connected to a RailBox data logger (EMS Brno, Czech Republic) and placed into the substrate of the pots with selected seedlings (three pots per each combination of ecotype and treatment, i.e. 18 in total), with the probe extending from the substrate surface down to a depth of 70 mm. Values of soil moisture were recorded at 1 h intervals. Gravimetric calibration of the substrate was performed to convert the measured electrical values to volumetric water content (VWC) values. The fully saturated substrate was allowed to dry completely under periodic weighing, after which a calibration curve was constructed. In parallel, soil water potential was measured in three replicates using Delmhorst Gypsum blocks with datalogger (MicroLog SP3, EMS Brno, Czech Republic) to determine the permanent wilting point (PWP). The soil water content corresponding to the permanent wilting point (PWP, at a soil water potential of –1.5 MPa) was calculated as 0.204 % VWC.

### Seedling growth and needle functional traits

The length of the terminal shoot was recorded with a hand-held meter starting the second bud stage according to the phenological stage of budburst in Scots pine [69].

Destructive needle sampling for the laboratory analyses was performed three times during the experiment: at the end of the water stress treatment 24 June (DAT 78), after two weeks of the recovery 15 July (DAT 99) on the previous-year needles (Needle-age-class; NAC2) and at the end of the experiment 15 September (DAT 161) on the current-year needles (NAC1). Sampled needles were used for the assessment of needle biophysical traits: chlorophyll a + b content, water content, leaf mass per area (LMA) and anatomical traits described in the Sect. "Needle anatomy".

Two complimentary needle samples were collected from each seedling for destructive, biochemical and biophysical analyses. Photosynthetic pigments (total chlorophyll a + b = Chl a + b; total carotenoids = Car) were extracted in the dark at 4 °C for seven days using N,N-dimethylformamide [70]. The content of photosynthetic pigments was determined by spectrophotometer (Evolution 201, Thermo Fisher Scientific, Waltham, MA, USA). Pigment concentrations were calculated according to the equations given in [71] and related to dry weight. Another set of needle samples was used to assess fresh and dry weight and to calculate water content as equivalent to weight reduction after drying of the needles. Projection needle area of the fresh needles was acquired by scanning (EPSON Perfection V600 Photo scanner with upper lamp), resolution 800dpi, and was analysed in ImageJ software (version 1.54f). From the dry weight and leaf area, LMA was calculated.

### Needle anatomy

**Fig. 2:**
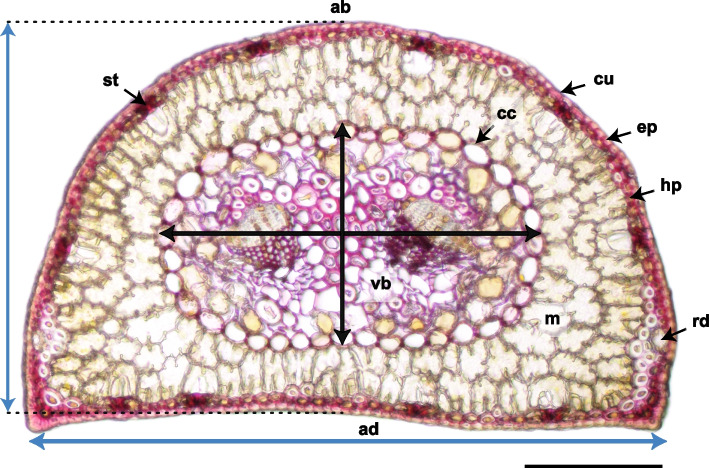
Scots pine needle cross-section. Needle abaxial side (ab), needle adaxial side (ad), cuticle (cu), epidermis (ep), lignified hypodermis (hp), arm-shaped palisade cells of mesophyll (m), central cylinder (cc), vascular bundle (vb), stoma (st), resin duct (rd). Blue arrows correspond to maximum cross-section thickness (vertical), measured in the middle of the needle and width (horizontal), black arrows correspond to central cylinder (including endodermis) thickness (vertical) and width (horizontal). Free-hand section, phloroglucinol—HCl staining (lignified cell walls stain cherry red). The bar corresponds to 200 µm

Needle anatomical traits were quantitatively assessed on cross-section micrographs. Fresh needles were fixed in an FAA solution composed of 70% ethanol, formalin, and glacial acetic acid mixed in a volumetric ratio of 18:1:1 (v:v:v). Fixed needle samples were cut using hand microtome to a section thickness of about 80 μm, stained with phloroglucinol—HCl to detect lignin in red coloured cell walls [72], and photographed using an Olympus BX40 microscope equipped with a Canon EOS100D camera. The analysed parameters were the needle cross-section area (Fig. 2), thickness and width of the cross-section and central cylinder. The proportion of epidermis and hypodermis, mesophyll, central cylinder and resin ducts on a needle cross-section area were determined as percentage of the total area. All measurements were accomplished in ImageJ.

### Chlorophyll fluorescence measurements

The chlorophyll fluorescence measurements started the fifth week of the drought treatment by imaging fluorometer Handy FluorCam FC 1000-H (Photon Systems Instruments, Drásov, CZ) using the built-in “Quenching protocol”. Seedlings were adapted to darkness for 30 min in a darkened room where all measurements were taken. A bunch of needles still attached to the shoot were put to the leaf clip of the device and subjected to the 192 s long quenching protocol, starting with saturation blue light pulse (455 nm; 4,900 μmol photons m^−2^ s^−1^), following five actinic light phases with respective saturation flashes and after following three dark relaxation phases again interrupted with saturation pulses (Supplementary Figure S1). Using FluorCam 7 software (Photon Systems Instruments, Drásov, CZ) the image was pre-processed, well focused and not noisy regions were selected for fluorescence parameters (Table 1) calculation (PSI 2019, FluorCam operation manual). Following fluorescence parameters were used for comparison between ecotypes among the treatments: QY_max_ (maximum quantum yield of dark-adapted PSII), QY L1 (quantum yield during the light adaptation phase), F_V_/F_M_ Lss (maximum PSII efficiency in light adapted steady state), NPQ Lss (steady-state non-photochemical quenching in light) [73], and Rfd (the radio of fluorescence decrease from the Fm to steady state) used as a vitality index [74].

**Table 1:** Monitored chlorophyll fluorescence parameters. The left column contains the observed parameter, the middle column contains the formula from which the parameter is calculated, and the right column of the table describes the process that the parameter defines. The measuring protocol consists of several (n) light (L) and dark (D) phases. For the graphical representation of the protocol see the Supplementary Figure S1, adapted from [75]

Parameter	Formula	Description
QY_max_ (F_V_/F_M_)	F_V_/F_M_	maximum PSII quantum yield in dark-adapted state
F_V_/F_M_ L*n*	(F_M_ L*n*—F_0_ Lss)/F_M_ L*n*	PSII quantum yield in light-adapted state
F_V_/F_M_ Lss	(F_M_ Lss—F_0_ Lss)/F_M_ Lss	PSII quantum yield in light-adapted steady-state
F_V_/F_M_ D*n*	(F_M_ D*n—*F_0_ Lss)/F_M_ D*n*	PSII quantum yield in dark-adapted stated
QY L*n*	(F_M_ L*n*—F_t_ L*n)*/F_M_ L*n*	PSII quantum yield induced in light outside the saturation pulse
QY Lss	(F_M_ Lss—F_t_ Lss)/F_M_ Lss	steady-state PSII quantum yield in light outside the saturation pulse
QY D*n*	(F_M_ D*n—*F_t_ D*n)*/F_M_ D*n*	PSII quantum yield relaxing in dark outside the saturation pulse
NPQ Lss	(F_M_—F_M_ Lss)/F_M_ Lss	steady-state non-photochemical quenching in light
Rfd Lss	(F_P_—F_t_ Lss)/F_t_ Lss	empiric parameter used to assess plant vitality; higher the values, higher vitality

### Spectral reflectance measurements, preprocessing and vegetation indices calculation

The reflectance of each seedling was assessed twelve times from the 22 of April (DAT 15) to the 15 July (DAT 99),2021. Shoot level reflectance was acquired on the fully developed, previous-year shoots (NAC2) (Supplementary Figure S2). The position of spectral measurements was kept constant for each seedling during the experiment, to ensure that the shoots were measured from the same position and direction. During each spectral measurement, an RGB image of the spectroradiometer field of view was taken to calculate the ratio of dark background to seedling shoot.

Individual seedling level reflectance was acquired by ASD FieldSpec4 Wide-Res spectroradiometer (ASD Inc., Boulder, CO, USA) using a range 350–2,500 nm and optical cable with field of view 25° with the distance of 24 cm from the seedling. Optical cable was used without the cosine or lenses. Two halogen lamps with the stable intensity for spectral range 350–2,500 nm (ASD Illuminator Reflectance Lamp, ASD Inc., Boulder, CO, USA) were used as the light source, with the angle 60° and the distance of the 50 cm from the seedling. Spectral measurement was performed in a dark room; seedling was placed in a black box painted by non-reflected black colour (NEXTEL VELVET COATING 811–21). Reflectance of this black background was used for the spectral correction and reflectance of the white panel with 99% of the reflectivity was used as spectral reference each 10 min. Reflectance measurements were recorded from 25 scans for each seedling and from 100 scans for the white reference.

#### Spectra correction for seedling and background signal ratio

The proportion of spectroradiometer´s field of view occupied by the seedling was not uniform due to the variability in crown architecture (branching, needle angle) and needle density among seedlings. The raw spectral curves had to be corrected for different signal ratio from the seedling and black background. The more background was in the field of view (FOV) of the fibre optic cable, the lower the measured radiometric values were in all bands. For this reason, it was necessary to adjust the measured values to obtain the spectral curve of the pine itself and to eliminate the influence of the black background.

Each seedling was photographed with a mobile phone fixed below the pistol grip with the optical cable. Before starting the measurements, a sheet of paper with a ruler was placed to the same distance (24 cm) from the optical cable as later the seedlings, and a calibration photograph with indication of the central point of the optical cable field of view was taken. Considering 25° FOV of the fibre optic cable and distance from the object (calibration sheet), the circle with the radius of 5.3 cm was set as an area of spectral measurement. On each seedling´s photo the exact position from where the signal entered the optical cable was determined and used for further processing (Supplementary Figure S2).

To separate black background pixels from seedling pixels in the photographs, we used a timesaving and computationally simple supervised k-th Nearest Neighbour pixel classification [76]. The classifier was trained on a dataset created from five photos of pine trees, containing a total of 126,150 pixels. Two classes were defined: pine pixels and black background pixels. The trained classifier was then applied to each seedling photo. Within the spectroradiometer’s field of view, the ratio of these two classes was calculated, providing the percentage of seedling and black background in the measured signal.

In the last step, an adjustment of the reflectance values based on the Linear Spectral Unmixing method was performed [77]. The resulting signal is assumed to be the sum of the reflectance values of several surfaces, and the weights of these values are assigned according to the cover percentage of the surface. It is based on the following formula:

1 $$I\left(\lambda \right)={C}_{1}*{R}_{1}\left(\lambda \right)+{C}_{2}*{R}_{2}\left(\lambda \right)$$

where I(λ) is the resulting reflectance at a certain wavelength, C_1_ and C_2_ are the percentages of certain surfaces, and R_1_ (λ) and R_2_ (λ) are the reflectance of certain surfaces at a certain wavelength. In the present experiment, I(λ) was the reflectance measured by the optical cable, C_1_ was the percentage of seedling in the field of view, C_2_ was the percentage of black background in the field of view, R_1_ (λ) was the reflectance of seedling at a certain wavelength, and R_2_ (λ) was the reflectance of black background at a certain wavelength. The reflectance of the homogeneous black background alone was measured 4 times each measurement day. From these measurements, the average value of the reflectance of the black box was calculated. The reflectance of the seedling in particular wavelength was calculated using Eq. 2. The resulting spectral curves of individual seedlings were obtained performing the above-described procedure in Matlab (version R2019a). Further analyses of the spectra were performed on the resulting reflectance values:

2 $${R}_{1}\left(\lambda \right)=\frac{I\left(\lambda \right)-{C}_{2}*{R}_{2}\left(\lambda \right)}{{C}_{1}}$$

#### Vegetation indices PRI and REP

The optical vegetation indices were calculated following the method of [66]. The photochemical reflectance index (PRI) was calculated as follows: as:

3 $$PRI=\frac{{R}_{531}-{R}_{570}}{{R}_{531}+{R}_{570}}$$

where R is the reflectance in specific wavelength. As the reflectance in 531 nm correlates positively with the epoxidation state of xanthophyll cycle pigments [78], lower index values correspond to more deepoxidation state and enhanced non-photochemical quenching processes.

The red edge position (REP) was estimated using a two-step calculation procedure based on linear four-point interpolation [79] applied to four wavebands (670, 700, 740, and 780 nm):

4 $$REP=700+40\left(\frac{{R}_{re}-{R}_{700}}{{R}_{740}-{R}_{700}}\right)$$

where calculation of the reflectance at the inflexion point (R_re_) was:

5 $${R}_{re}=\left({R}_{670}+{R}_{780}\right)/2$$

### Statistical analyses

Differences between ecotypes, treatments and their interactions were performed in R [80] and the ASReml for R [81].

We examined the variation in needle functional traits among control, mild drought and drought stressed plants using a univariate linear mixed model:

6 $$\begin{aligned} y&=1\mu +{X}_{1}{\beta }_{s}+{X}_{2}{\beta }_{t}+{X}_{3}{\beta }_{d}+{X}_{4}{\beta }_{st}\\&+{X}_{5}{\beta }_{td}+{X}_{6}{\beta }_{sd}+{X}_{7}{\beta }_{std}+{Z}_{1}b+e, \end{aligned}$$

where ***y*** corresponds to the phenotypic value; μ is the overall mean effect; *β*_*s*_ is a fixed effect of the ecotypes (upland and lowland); *β*_*t*_ is a fixed effect of the treatment (control, mild drought and drought); *β*_*d*_ is a fixed effect of the DAT, *β*_*st*_ is the fixed interaction effect between ecotype and treatment, *β*_*td*_ is the fixed interaction effect between treatment and DAT, *β*_*sd*_ is the fixed interaction effect between ecotype and DAT, *β*_*std*_ denotes the interaction effect among ecotypes, treatments and DAT; *bd* denotes the vector of block × DAT interaction effects, with:

7 $$bd\sim \mathcal{N}\left(0,{\sigma }_{\mathrm{bd}}^{2}{\mathbf{I}}_{\mathrm{bd}}\right),$$

I_bd_ denotes the identity matrix for the block × DAT random effect, and σ^2^_bd_ is the associated variance component. *X*_*i*_ are respective incidence matrices associated with fixed effect, *Z* is the incidence matrix associated with the random effect; and *e* is the random error term, with:

8 $$e\sim \mathcal{M}\mathcal{V}\mathcal{N}\left(0,{\mathbf{I}}_{\mathbf{n}}\otimes \mathbf{R}\right),$$

where *R* is a 19 × 19 matrix of heterogeneous first-order autoregressive (AR1) over DAT, allowing the correlation to vary with time, and *I*_*n*_ is an identity matrix identifying each tree.

Wald test was implemented to evaluate the fixed effects of population, treatment, and their interaction on all measured traits [81]. Given the limited number of traits and their strong biological correlation, we are presenting unadjusted p-values of the fixed effect.

Multiple comparisons among factors were calculated using predictPlus function (asremlPlus package), p-values were then FDR adjusted by Benjamini–Hochberg method using the base R p.adjust function. Corresponding effect sizes were calculated as Cohen’s *d*.

Redundancy analysis (RDA) was used to assess the effects of ecotype and treatments on NAC2 in final stress phase (DAT 78) and after two weeks of recovery (DAT 99), and at the end of the recovery period (DAT 161) on NAC1. Standardized values of selected needle functional traits were analysed using the *vegan* package [82] in R [80]. Response variables were z-score standardized prior to analysis, and model significance was tested by permutation.

## Results

### Water availability during the drought stress and recovery phase

**Fig. 3:**
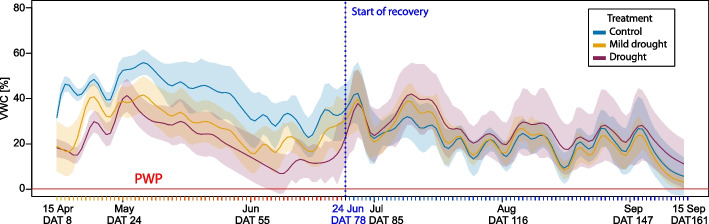
Volumetric water content (VWC) during the experimental stress from 15 April (DAT 8) to 24 June (DAT 78) and recovery period from 24 June to 15 September (DAT 161). The curves represent the mean of VWC for each treatment (Control – C – blue; Mild drought – MD – yellow; Drought – D – red), shadow in respective colour represents the standard deviation of VWC for each treatment. The vertical blue dashed line marks the beginning of the recovery period (24 June). The horizontal red line marks the permanent wilting point (PWP = 0,204). *n* = 6 per treatment

The volumetric water content (VWC) was recorded during the experimental period (Fig. 3). Although the irrigation was not corrected for water consumption on the level of individual trees, the difference in VWC among the treatments was achieved. However, even under the more severe irrigation reduction (D), the substrate never reached the permanent wilting point. The average VWC for differently treated groups is shown in Fig. 3. Although VWC fluctuated remarkably, the soil moisture gradient among the control (C), mild drought (MD) and drought (D) treatments was present in the stress period and diminished during the recovery phase of equal irrigation. The higher mean values of VWC in drought-treated seedlings during the recovery phase may result from their acclimation to lower water availability and reduction in water consumption.

### Seedling growth and mortality

**Fig. 4:**
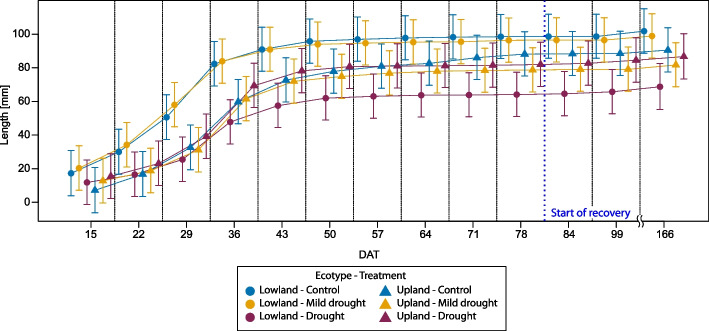
Terminal shoot length. X-axis: days after transplantation (DAT 15–166). Weakly measurements during the stress and recovery period until the DAT 84, later in the recovery period the measurement interval was longer and irregular. The vertical blue dashed line marks the beginning of the recovery period at 24th June (DAT 78). Symbols represent the average predicted values for the terminal shoot length of upland and lowland Scots pine ecotypes with different water treatments (different colours), obtained by univariate linear mixed model with predicted 95% confidence interval. *n* = 15 per each combination of ecotype and treatment

The terminal shoot length was monitored since the beginning (DAT 8) of the experimental treatment (Fig. 4). In both ecotypes the growth of terminal shoot was most pronounced between the third and fourth week of the experiment (DAT 29 and 36). Statistical analysis showed no significant effects of ecotype and treatment alone during the stress period, however, three-factor interaction among treatment, ecotype and DAT was significant. Compared to that the effect of treatment and also interaction of treatment and ecotype was significant in the recovery period (summed up in Supplementary Table S1). There was an apparent trend for different growth reaction of the two pine ecotypes. In the lowland ecotype the terminal growth was reduced only under the drought treatment. Contrastingly, in the upland ecotype, the treatment effect was not conclusive, and the shoot length was statistically unaffected (Fig. 4). To sum up, the drought treatment resulted in notable reduction in final terminal shoot length in lowland ecotype, while the upland ecotype was not responsive.

The seedling mortality after the whole experimental period (including drought and recovery) differed between the ecotypes. The lowland ecotype seedling mortality was 13 %, 0 %, and 47 % in control, mild drought, and drought, respectively. The upland ecotype seedling mortality was 13 %, 0 %, and 29 % in control, mild drought, and drought, respectively. That is, the only difference in reaction of ecotypes was found in the drought treatment when the lowland ecotype exhibited one third higher seedling mortality compared to the upland ecotype. Differences between the upland and lowland drought treatment 18 % correspond to only 2–3 individuals, which limits the statistical reliability of comparisons between ecotypes. In addition, the 13% mortality observed in the control treatment indicates that part of the mortality was not caused by drought.

### Needle functional traits

**Fig. 5:**
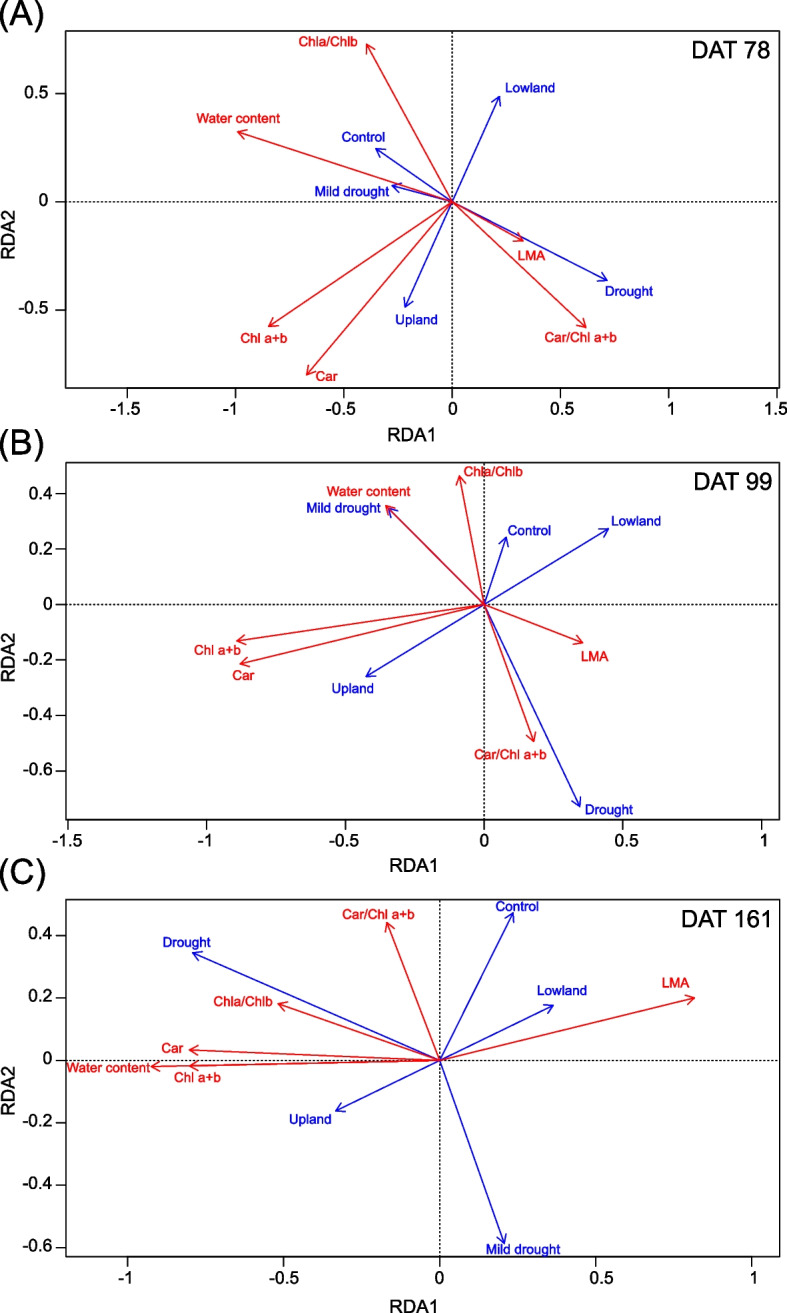
Redundancy analysis (RDA) ordination diagrams showing relationships between physiological traits (red arrows) and constraining variables (Ecotype, Treatment; blue arrows) in the studied seedlings at three time points:(**A**)DAT 78,(**B**)DAT 99, and (**C**) DAT 161. Arrow length and direction indicate the strength and orientation of correlations with the canonical axes. The relative positions of arrows reflect gradients in trait responses to the constraining variables

At the end of the stress period (DAT 78) on NAC2, RDA analysis showed that the predictors ecotype and treatment explained 23.8 % of the variance in the data (R^2^ = 23.8 %) (Fig. 5 A). The overall model was signific*ant (*p = 0.001), with both predictors contributing significantly (ecotype *p* = 0.001; treatment *p* = 0.001). The first two canonical axes together accounted for 97.2 % of the variance captured by the model (RDA1 = 56.9 %, RDA2 = 40.2 %).

After two weeks of rewatering (DAT 99) on NAC2, RDA analysis showed that ecotype and treatment together explained 12.1 % of the variance in the data (R^2^ = 12.1 %) (Fig. 5 B). The decrease in variance explained by the predictors suggest regeneration in analysed traits after rewatering. The overall model was significant (*p* = 0.003), with both predictors contributing significantly (ecotype *p* = 0.006; treatment *p* = 0.047). The first two canonical axes accounted for 98 % of the variance captured by the model (RDA1 = 72.1 %, RDA2 = 25.9 %).

At the end of the recovery period (DAT 161) on NAC1, RDA analysis showed that ecotype and treatment together explained 16.9 % of the variance in the data (R^2^ = 16.9 %) (Fig. 5 C). The overall model was significant (*p* = 0.002), with both predictors contributing significantly (Ecotype *p* = 0.006; Treatment *p* = 0.005). The first canonical axis alone accounted for 90.9 % of the variance explained by the model.

The most prominent and stable outcome of the RDA was the clear separation of samples by ecotype. In contrast, the responses of individual physiological parameters varied depending on the measurement date and the age of needles. These differences will be examined in detail in the following sections, where the results of individual parameters are presented.

**Fig. 6:**
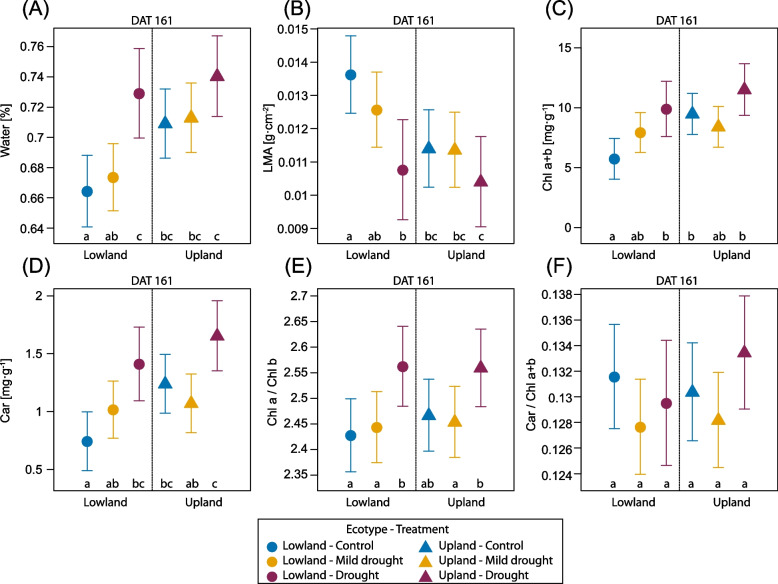
Selected functional traits of current-year needles (NAC1) at the end of the growing season (DAT 161). Water content (**A**), leaf mass per area (**B**), content of chlorophyll *a* + *b* (**C**) and carotenoids (**D**), ratio of chlorophyll *a* and chlorophyll *b* (**E**), ratio of carotenoids and chlorophyll *a* + *b* (**F**). Symbols represent the average predicted values for functional leaf traits of upland and lowland Scots pine ecotypes with different water treatments (different colours), obtained by univariate linear mixed model with predicted 95% confidence interval. Different letters indicate significant differences (α = 0.05) between treatment × ecotype combinations. *n* = 8–15 per each combination of ecotype and treatment

Water content in previous-year needles (NAC2) decreased significantly under the drought treatment in comparison to the control in both ecotypes. After two weeks of recovery, the water content of the needles was fully restored and the difference between treatments was negligible (Table 2). Water content in current-year needles (NAC1) was evaluated only at the end of the growing season and was affected by the ecotype and treatment. Upland ecotype had generally higher needle water content than lowland ecotype in response to drought treatment during the needle development in the current year. Surprisingly, the drought treated seedlings of both ecotypes had significantly higher water content than control and mild drought treated ones (Fig. 6 A).

**Table 2:** Selected functional traits (Water, LMA, Chl a + b, Car, Chla/Chlb, Car/Chl a + b) of previous-year (NAC2) and current-year needles (NAC1). Sampling of NAC2 needles was done at the end of the stress period (DAT 78) and after two weeks of rewatering (DAT 99), sampling of NAC1 needles was done at the end of recovery period (DAT 161). P-values associated with fixed factors: Treatment, Ecotype and interactions between the Treatment and Ecotype. Significant p-values (< 0.05) are marked in bold. *n* = 24–28 per each combination of ecotype and treatment for NAC2 needles and *n* = 8–15 per each combination of ecotype and treatment for NAC1 needles

	***p*-value**					
	**Water**			**LMA**		
	**NAC2**		**NAC1**	**NAC2**		**NAC1**
**Factor**	**DAT 78**	**DAT 99**	**DAT 161**	**DAT 78**	**DAT 99**	**DAT 161**
Treatment	** < 0.001**	0.101	** < 0.001**	**0.022**	**0.047**	**0.006**
Ecotype	0.136	0.094	** < 0.001**	0.900	0.067	**0.003**
Treatment:Ecotype	0.636	0.309	0.362	0.824	**0.023**	0.306
	**Chl a + b**			**Car**		
	**NAC2**		**NAC1**	**NAC2**		**NAC1**
**Factor**	**DAT 78**	**DAT 99**	**DAT 161**	**DAT 78**	**DAT 99**	**DAT 161**
Treatment	** < 0.001**	**0.0298**	**0.002**	**0.009**	0.056	** < 0.001**
Ecotype	** < 0.001**	** < 0.001**	**0.003**	** < 0.001**	** < 0.001**	**0.006**
Treatment:Ecotype	0.775	0.499	0.091	0.929	0.619	0.123
	**Chla/Chlb**			**Car/Chl a + b**		
	**NAC2**		**NAC1**	**NAC2**		**NAC1**
**Factor**	**DAT 78**	**DAT 99**	**DAT 161**	**DAT 78**	**DAT 99**	**DAT 161**
Treatment	** < 0.001**	**0.003**	** < 0.001**	** < 0.001**	**0.019**	0.087
Ecotype	0.124	0.874	0.512	0.573	0.798	0.624
Treatment:Ecotype	0.968	0.436	0.432	0.313	0.668	0.430

Leaf mass per area (LMA) of NAC2 needles did not differ between ecotypes but responded to drought treatments in stress period (Table 2). After two weeks of re-watering, differences between treatments were still significant. LMA of NAC1 needles developed during the reduced water availability was evaluated in September after the recovery period. Upland and lowland ecotype responded differently to water shortage during the needle development. The lowland ecotype showed significantly lower LMA for seedlings under the drought treatment compared to the control. In contrast, the response in LMA to water deficit was less evident in the upland ecotype for seedlings under the drought treatment. The upland ecotype showed remarkably lower LMA compared to the lowland ecotype, including the plants in control conditions (Fig. 6 B).

There was a significant effect of ecotype and treatment on chlorophyll a + b content in NAC2 needles after the water stress period and during the recovery phase (Table 2). The chlorophyll a + b content in NAC1 needles was also affected by treatment, there are apparent differences in drought response of upland and lowland ecotypes (Fig. 6 C). Both ecotypes showed signs of compensatory response in (NAC1) needles developed under water deficit: drought-treated seedlings showed higher chlorophyll content at the end of the growing season (i.e. after two months of regeneration; compared to controls). The content of the protective pigments – carotenoids – showed a similar trend and was remarkably higher in seedlings treated with drought compared to control and mild drought (Fig. 6 D). Throughout the experiment, there was a significant difference in the chlorophyll a and b ratio between the treatments for both ecotypes, at the end of the experiment, the ratio was higher for NAC1 needles in the drought treatment compared to the other treatments (Fig. 6 E). In contrast, a significant difference in the ratio of carotenoids and chlorophylls was found only in the NAC2 needles (Table 2).

### Needle anatomy

**Fig. 7:**
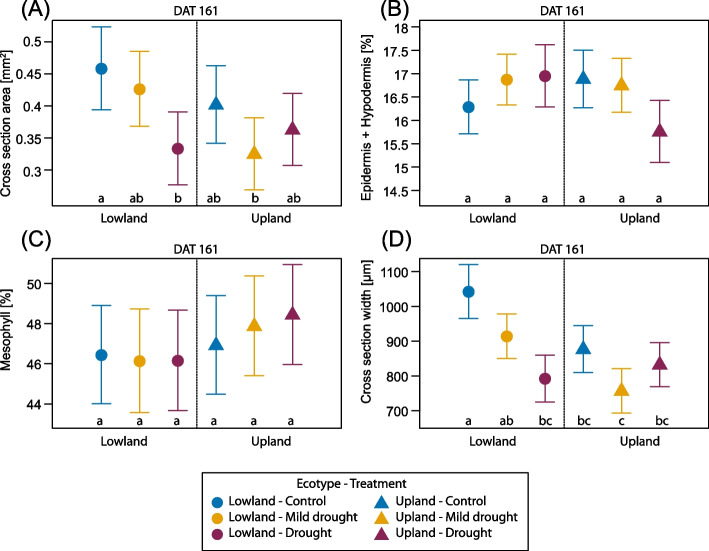
Selected needle anatomical traits evaluated on the current-year needles (NAC1) at the end of the growing season (DAT 161). Cross-section area (**A**), Epidermis and hypodermis proportion on needle cross-section area in percentage (**B**), mesophyll proportion on needle cross-section area in percentage (**C**), cross-section width (**D**). Symbols represent the average predicted values for needle anatomical parameters of upland and lowland Scots pine ecotypes with different water treatments (different colours), obtained by univariate linear mixed model with predicted 95% confidence interval. Different letters indicate significant differences (α = 0.05) between treatment × ecotype combinations. n = 15 per each combination of ecotype and treatment

Current-year needle (NAC1) anatomy was quantitatively evaluated at the end (DAT 161) of the growing season on needles that developed under induced drought and subsequent recovery phase. All the anatomical parameters, which showed significant response to the treatment or differed between ecotypes, are shown in the Table 3. Needle cross-section area and width showed significant reduction due to drought treatment. The effects of the ecotypes and the interaction of treatment and ecotype were also significant. The lowland ecotype achieved higher values for both parameters than the upland ecotype, simultaneously seedlings in the control treatment of the lowland ecotype reached higher values than needles treated with drought (Fig. 7). The width of the central cylinder with endodermis showed the same pattern. Interestingly, the less the watering, the higher the proportion of epidermis and hypodermis in needle cross-section for the lowland ecotype and the opposite for the upland ecotype.

**Table 3:** Effect of ecotype and drought treatment on needle anatomical traits at the end of the growing season (DAT 161). % refers to the proportion in percentage of the whole cross-section area. P-values associated with fixed factors: Treatment, Ecotype and interactions between the Treatment and Ecotype. Significant p-values (< 0.05) are marked in bold. n = 15 per each combination of ecotype and treatment

	***p*-value**				
**Factor**	**Cross-section area (mm**^**2**^**)**	**Epidermis and hypodermis %**	**Mesophyll %**	**Resin duct %**	**Cross-section width (µm)**
Ecotype	**0.009**	0.324	0.807	0.100	** < 0.001**
Treatment	**0.032**	0.494	0.071	**0.025**	** < 0.001**
Ecotype:Treatment	**0.022**	**0.019**	0.640	0.443	**0.002**

### Chlorophyll fluorescence parameters

**Fig. 8:**
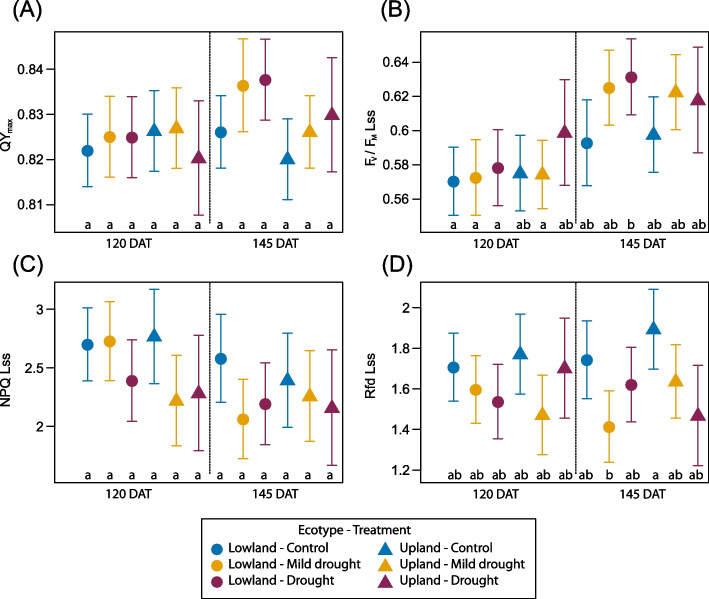
Selected chlorophyll fluorescence parameters evaluated on the current-year needles (NAC1) calculated from the Quenching protocol – QY_max_ (**A**), F_V_/F_M_ Lss (**B**), NPQ Lss (**C**), Rfd Lss (**D**). Both dates (DAT 120 and 145) are from the rewatering period. Symbols represent the average predicted values for chlorophyll fluorescence parameters of upland and lowland Scots pine ecotypes with different water treatments (different colours), obtained by univariate linear mixed model with predicted 95% confidence interval. Different letters indicate significant differences (α = 0.05) between treatment × ecotype x DAT combinations. n = 3–6 per each combination of ecotype and treatment

The drought response of chlorophyll fluorescence parameters (Table 1) acquired from the quenching analysis was evaluated for both previous (NAC2) and current-year (NAC1) needles of Scots pine. The effects of ecotype, treatment, time, and their interactions are summed up in Table 4. In general, for NAC2 needles, the only two suitable parameters for detecting current exposure to drought at the treatment or ecotype level, were QY Lss and QY L1. For retrospective detection of drought on NAC1 needles, parameters F_V_/F_M_ Lss, QY L1, QY Lss and Rfd Lss seemed to be suitable. Both ecotypes exhibited a trend of increase in QY_max_ in both drought treatments compared to control at the end of recovery period (DAT 145) (Fig. 8 A). This pattern corresponded with higher chlorophyll content in drought-treated seedling needles NAC1. After two months of recovery (DAT 145) the upland ecotype showed lower values of QY_max_ but greater variability compared to the lowland ecotype, in both mild drought and drought-treated seedlings. A similar trend of lower primary photosynthetic activity under control conditions at DAT 145 was observed in the parameter F_V_/F_M_ Lss (Fig. 8 B). Increasing NPQ values in NAC2 needles (Supplementary Table S3) indicate increased activation of protective mechanisms against PSII damage during the stress period. However, during the recovery period, NPQ values remained higher in NAC2 needles in both ecotypes, particularly in the upland one. Despite this, seedlings of the upland ecotype showed demonstrably more efficient primary photosynthetic processes during drought treatment compared to the lowland ecotype, based on the parameter Rfd. NAC1 needles achieved the highest values of both NPQ and Rfd during recovery under control conditions (Fig. 8 C,D). This suggests that needles developed under water scarcity may be unable to achieve an optimal balance between protection and efficiency of the photosynthetic apparatus. Drought probably limited the regenerative capacity of PSII in NAC1 needles, as reflected by significantly lower values of both parameters in comparison to control seedlings.

**Table 4:** Selected chlorophyll fluorescence parameters calculated from the quenching protocol in stress and recovery period. QY_max_ – the maximum quantum fluorescence yield of dark-adapted needles, F_V_/F_M_ Lss—PSII maximum efficiency of light adapted sample in steady-state, QY L1 – PSII quantum yield induced in light outside the saturation pulse, QY Lss – PSII quantum yield in steady-state in light outside the saturation pulse, NPQ Lss – Steady-state non-photochemical quenching, Rfd Lss – fluorescence drop ratio on steady state. Six measurements were taken on previous-year needles (NAC2) during the stress period and two measurements at the beginning of the recovery period; two measurements were taken on the current-year needles (NAC1) during the recovery period. Significant p-values (< 0.05) are marked in bold. *n* = 3–6 per each combination of ecotype and treatment

	***p*-value**					
	**QY**_**max**_			**F**_**V**_**/F**_**M**_** Lss**		
	**NAC2**		**NAC1**	**NAC2**		**NAC1**
**Factor**	**Stress**	**Recovery**	**Recovery**	**Stress**	**Recovery**	**Recovery**
**Treatment**	0.746	0.100	0.234	0.121	0.536	**0.034**
**Ecotype**	0.054	0.628	0.285	0.151	**0.005**	0.828
**DAT**	** < 0.001**	0.223	0.094	**0.006**	0.571	** < 0.001**
**Treatment:Ecotype**	0.856	0.354	0.758	0.248	0.355	0.962
**Treatment:DAT**	0.210	0.987	0.136	** < 0.001**	0.154	0.117
**Ecotype:DAT**	**0.004**	0.726	0.062	**0.002**	0.369	0.395
**Treatment:Ecotype:DAT**	0.927	0.921	0.780	0.067	0.527	0.501
	**QY L1**			**QY Lss**		
	**NAC2**		**NAC1**	**NAC2**		**NAC1**
**Factor**	**Stress**	**Recovery**	**Recovery**	**Stress**	**Recovery**	**Recovery**
**Treatment**	0.071	**0.021**	**0.003**	**0.003**	0.344	**0.028**
**Ecotype**	** < 0.001**	** < 0.001**	0.812	0.812	0.488	0.661
**DAT**	**0.008**	0.923	0.417	0.417	**0.002**	** < 0.001**
**Treatment:Ecotype**	**0.021**	0.803	0.083	0.083	0.427	0.675
**Treatment:DAT**	** < 0.001**	**0.011**	**0.005**	**0.005**	0.072	0.243
**Ecotype:DAT**	0.491	0.220	**0.017**	**0.017**	0.455	0.891
**Treatment:Ecotype:DAT**	0.052	**0.028**	**0.029**	**0.029**	0.055	0.594
	**NPQ Lss**			**Rfd Lss**		
	**NAC2**		**NAC1**	**NAC2**		**NAC1**
**Factor**	**Stress**	**Recovery**	**Recovery**	**Stress**	**Recovery**	**Recovery**
**Treatment**	0.132	0.369	0.062	0.149	0.470	**0.003**
**Ecotype**	0.677	0.058	0.433	0.104	**0.010**	0.320
**DAT**	**0.045**	**0.025**	** < 0.001**	**0.009**	0.717	0.973
**Treatment:Ecotype**	0.147	0.789	0.927	**0.026**	0.814	0.818
**Treatment:DAT**	**0.008**	0.840	0.565	**0.009**	0.147	0.391
**Ecotype:DAT**	0.501	0.355	0.303	**0.002**	0.122	0.236
**Treatment:Ecotype:DAT**	0.717	0.176	**0.038**	** < 0.001**	0.837	** < 0.001**

### Shoot spectral reflectance for drought detection: indices PRI and REP

Vegetation indices PRI and REP for lowland and upland ecotypes of Scots pine seedlings were examined during the drought stress period (10 measuring weeks; DAT 15–78) and the recovery period (2 measuring weeks; DAT 84 and 99) (Supplementary Figure S3, S4).Vegetation indices PRI and REP calculated for previous-year shoots (NAC2) did not consistently reflect drought effects (Table 5).

**Table 5:** Effect of ecotype, drought treatment, time, and their interactions of the photochemical reflectance index (PRI) and the red edge position (REP), calculated based on spectra measured on previous-year shoots (NAC2). Significant p-values (< 0.05) are marked in bold. *n* = 25–30 per each combination of ecotype and treatment

	***p*-value**			
	**PRI**		**REP**	
**Factor**	**Stress**	**Recovery**	**Stress**	**Recovery**
Treatment	0.299	0.234	0.376	0.933
Ecotype	** < 0.001**	** < 0.001**	**0.04**	**0.007**
DAT	** < 0.001**	** < 0.001**	** < 0.001**	** < 0.001**
Treatment:Ecotype	** < 0.001**	** < 0.001**	**0.027**	0.352
Treatment:DAT	0.689	**0.023**	** < 0.001**	0.872
Ecotype:DAT	** < 0.001**	0.127	** < 0.001**	0.588
Treatment:Ecotype:DAT	0.834	0.152	0.555	**0.001**

REP showed a gradual increase during the experiment in all treatments, likely reflecting accumulation of photosynthetic pigments during needle development. A decline occurred under drought at DAT 71 in all treatments, with partial recovery in the upland ecotype after rewatering. PRI showed no consistent drought-related pattern. These findings suggest that fully developed needles from previous season (NAC2) do not readily respond to drought in their optical properties and may not be suitable for drought resilience assessment using PRI and REP.

## Discussion

We observed phenotypic plasticity and ecotypic differentiation in growth reactions of a wide range of needle functional traits of *P. sylvestris* from two distinct Czech populations representing lowland and upland ecotypes in response to spring drought and subsequent recovery phase. We observed that different responses of lowland and upland ecotypes to drought could be to some extent detected by spectral reflectance of seedlings during the drought and recovery period.

### The upland ecotype does not reduce terminal growth and has better survival

Plant growth and survival depend on water availability, which affects phenology and biomass accumulation. In Scots pine seedlings, drought stress causes a shorter apical shoot length [27] and also affects reduction of the total aboveground biomass [16]. Our findings for the lowland ecotype align with the above-mentioned literature, while lack of significant treatment effect on shoot length in the upland ecotype under drought suggest greater resilience to water scarcity. Another response to the drought treatment is the reduction of the transpiration surface by needle shedding [83], but we did not observe this phenomenon systematically in our study. However, the current-year needles (NAC1) of both ecotypes showed smaller cross-section area and needle width under drought treatment, which indicate a reduction in both cell growth and assimilation area at the level of individual organs.

Higher allocation of biomass in needles of non-native *P. sylvestris* ecotypes could lead to increased transpiration rate, which would be maladaptive in dry environments and has been linked to higher mortality [84, 85]. In our study, we observed that the lowland ecotype exhibited higher mortality (47 %) under the drought treatment compared to the upland ecotype (29 %). This aligns well with its stronger reduction in investment to LMA (Fig. 5A), potentially lead to greater vulnerability under drought stress. Additionally, dry weight of seedlings and their height are the main determinants related to acclimation potential against drought mortality, which is quite specific within individual ecotypes of Scots pine [86]. In our study, the lowland ecotype showed greater height growth under control conditions but suffered significantly higher mortality under drought, this pattern should be viewed as a preliminary indication rather than a robust demonstration of greater drought resilience in the upland ecotype. We also note that, given the mild drought stress actually experienced by the upland seedlings, the observed differences in mortality (mild drought 0 % and drought 29 %) may reflect individual variation rather than a consistent ecotypic response. In contrast, the upland ecotype maintained a more equilibrate growth across treatments, which may contribute to its comparatively higher survival rate. Thus, we can conclude that based on main shoot growth and mortality, the upland ecotype showed more conservative (isohydric) water use, which resulted in better resilience to drought stress and recovery compared to lowland ecotype. However, in long-term the conservative water use may lead to nutrient imbalances resulting from transpiration stream and nutrient uptake reduction [87].

### Most needle traits show recovery after rewatering in both ecotypes

A key parameter for determining the efficiency of water use and the amount of light captured by the photosynthetic process is leaf size, which can be expressed as LMA [88, 89]. Among nine pine species from the circum-Mediterranean area, LMA negatively correlated with the precipitation in the warmest three-month period [90]. This result also confirmed Houmier et al. [91], suggesting that the relatively high LMA of hybrids between Aleppo pine (*Pinus halepensis* Mill.) and Calabrian pine (*Pinus brutia* Ten.) could be an advantage for thriving in marginal areas characterized by low rainfall and high solar radiation. However, at the intraspecific scale, long-term provenance experiments with Scots pine revealed no significant differences in LMA between regional populations, despite significant genetic intra-population variability [92]. Similarly, in our study, LMA did not differ between ecotypes in NAC2 needles, likely due to their development under non-stressful conditions in the previous growing season. However, in NAC1 needles developed under drought conditions, LMA for both ecotypes positively correlated with irrigation levels, indicating that water availability influences biomass allocation in needle tissue. Our results further support findings by Bhusal et al. [93], who reported a negative correlation between absolute leaf size and drought resistance. In our study, the upland ecotype, which exhibited lower LMA, also showed lower mortality, suggesting a potential link between these traits. Furthermore, the reduced LMA and shorter terminal shoots observed in drought-treated lowland seedlings align with findings by Bachofen et al. [27], who suggested that shorter and fewer shoots of pine species can enhance hydraulic safety by reducing the transpiring surface area. NAC1 needles of seedlings exposed to the drought treatment reached the highest water content values in both ecotypes (Supplementary Figure S5), which may indicate the achievement of hydraulic safety through improved water management. According to Rehschuh et al. [94] the recovery in needle water content indicates restoring of functional extraxylary needle tissues.

The content of photosynthetic pigments such as total chlorophylls and carotenoids are common indicators of stress in vascular plants. These pigments play a key role in photosynthesis, and their abundance allows an estimate of the photosynthetic capacity of a leaf or stand [95]. Except for the Car/Chl a + b ratio, all pigment traits showed higher levels in upland ecotype, which corresponds to high chlorophyll content positive association with better survival [30]. As expected, NAC2 needles in our study tended to decrease chlorophyll content with the drought treatment, consistently in response to *Pinus nigra* and *Pinus brutia* due to low rainfall and high summer temperatures [96]. Similarly to Qian´s study [43] we observed a recovered pigment content after rewatering in NAC2, and NAC1 needles had even higher chlorophyll and carotenoid values compared to control. Under drought stress, the primary function of carotenoids is protective and they play an important role in the survival of the plant [97, 98]. Similarly to other pine species [99, 100] we observed an increased ratio of carotenoids and chlorophyll a and b in the drought treatment of NAC2 needles at the end of stress period and during recovery. Our results suggest that Scots pine protects its photosynthetic apparatus more effectively under the drought treatment conditions and is characterized by lower sensitivity to photoinhibition compared to mild drought.

### The lowland ecotype had thinner needles under drought and invested more to non-photosynthetic tissues

A period of water deficiency can trigger anatomical plasticity in needles, leading to structural differences between needles developed under sufficient and insufficient water conditions, especially when it occurs in spring [101]. Studies focused on anatomical acclimation and plastic responses of pine needles show that one of the typical drought effects is a reduction in needle length and thickness [101, 102]. These changes represent an acclimation mechanism and lead to better water conservation by reducing the transpiring area [103]. Cross-section area and needle width were ecotype-specific traits and significantly decreased with reduced water availability in the lowland ecotype, while in the upland one were unaffected by the drought treatment applied in this study (Fig. 7) again indicating better drought resilience in the upland ecotype.

Differences in mesophyll volume fraction among pine species have been linked to differences in photosynthetic activity, as higher mesophyll volume fractions in needles are associated with increased photosynthetic capacity under higher irradiances [104]. Under stronger water deficit, the upland ecotype invested demonstrably less in the formation of epidermis, hypodermis and other non-photosynthetic tissues in needles, but the percentage of mesophyll increased slightly with the degree of water deficit, which is consistent with the results of [27]. The limited anatomical changes in needles observed in our study (Supplementary Figure S8) support the conclusion that structural acclimation to drought in Scots pine is relatively constrained [105].

### Neither ecotype suffered photoinhibition under drought

Damage to photosystem II (PSII) is one of the first manifestations of abiotic stress in plants [106] and its status can be monitored by chlorophyll a fluorescence [74, 107]. Analysis of chlorophyll fluorescence yield provides information on changes in the efficiency of photochemical processes and the ability to dissipate energy as heat (i.e. non-photochemical quenching), when light energy can no longer be efficiently used for photochemical processes [106, 108–110].

In our study, drought did not affect QY_max_, about 82 % of the energy absorbed by PSII was potentially available for photochemical processes in both ecotypes throughout the experiment (Supplementary Table S3), which is consistent with findings in Scots pine and Norway spruce reported by [111]. Thus, QY_max_ appears to be quite resistant to water limitation in juvenile Scots pine as it could hold stable high values (around 0.75) in seedlings with no irrigation for approximately 50 days [53]. Contrastingly, short-term summer drought resulted in a modest decrease in QY_max_ (from 0.83 to 0.70) in 3-year-old Scots pine seedlings [66]. The results of fluorescence measurements show that the ecotype and treatment, as well as their interaction, mainly affected the dark-to-light transition phase and light-acclimated steady state, i.e. the parameters QY L1, F_V_F_M_ Lss, and QY Lss (Table 4).

In our study, the upland ecotype demonstrates a better regeneration capacity than the lowland, as reflected by higher values of Rfd Lss, chlorophyll a and b and carotenoids during limited watering and subsequent regeneration, compared to the lowland ecotype. This supports the interpretation that the upland ecotype maintains greater photosynthetic potential under stress. Rfd Lss has been identified as a valuable indicator of leaf and tree vitality, with higher values reflecting increased photosynthetic activity [74], which aligns with our findings. In addition to the current physiological status of plants, chlorophyll fluorescence analysis can provide insights into phenotypic and genotypic variation of individual species or provenances [64, 112].

### Drought response evaluation based on needle optical properties

While REP showed gradual changes in time, especially under drought treatment and during recovery, its dynamics likely reflects long-term pigment adjustments or acclimation [113], rather than acute stress. Contrastingly to Miettinen et al.[66] showing PRI sensitivity to acute water stress and recovery, we did not observed any treatment-related pattern. The limited drought sensitivity of PRI and REP in our study likely results from their measurement on previous-year needles (NAC2), which responded less plastically than current-year needles (NAC1). These older needles may retain structural and pigment-related characteristics from the previous season, making them less suitable for detecting acute stress. This interpretation is further supported by the absence of LMA differences between ecotypes in NAC2 foliage, as discussed earlier. Early differences between treatments and ecotypes (already visible at DAT 15) suggests that these indices reflected initial physiological or genetic differences rather than actual drought response.

These findings emphasize the importance of selecting appropriate organs or tissue for spectral measurements. Our results, in agreement with the fluorescence-based indicators, suggest that NAC1 needles are more suitable for drought stress detection using spectral indices.

### Study limitations

Pot experiments simulating drought on tree seedlings, especially under meticulously controlled environments such as ventilated greenhouses or cultivation boxes, enable better control of the environmental conditions, significantly reducing residual variability and enhancing the precision of experimental outcomes. However, its ability to predict field performance of older trees is limited not only by different age and developmental period but also by “microcosm” pot limitation affecting the composition and performance of soil biota, particularly microorganisms. Future research should include long-term field trials on tree species under standardized conditions to verify practical relevance for older trees. A study by Forero et al. [114] on herbaceous species suggests that correlations between greenhouse and field performance can be weak or inconsistent. Such an experimental setting similar to the one used in this study has been frequently used for studies on physiological and biochemical leaf functional traits characterizing water stress of Pine species [16, 115]. Moreover, the greenhouse pot experiment setting resembles better nursery conditions for seedling screening. Valuable insights into Scots pine local adaptations and stronger generality of conclusions could be achieved by expanding the study to include additional ecotypes across broader environmental gradients (e.g. altitudinal, latitudinal, stand conditions) and by extending observations beyond the seedling and juvenile phase. However, this experiment provided valuable pilot insights that helped to shape the experimental design of a larger study conducted in 2022 [33], which incorporated multiple ecotypes, extended drought treatments, and genomic analyses to examine drought responses more comprehensively. These local adaptations are maintained through evolutionary trade-offs, where traits that enhance drought avoidance or drought tolerance in one habitat may reduce growth or carbon assimilation in another. Additionally, our study did not account for potential maternal effects or epigenetic modifications that could influence seedling drought responses independently of genomic variation, factors that may play important roles in short-term adaptive capacity and phenotypic plasticity across generations. Including detailed analyses of root architecture, as highlighted in previous study [116], could further improve our understanding of drought tolerance of temperate coniferous tree species. However, the seedlings were transplanted to the forest stand, which could enable to follow the drought legacy effect in the future, which is currently under our ongoing research. This was also the reason why the destructive analysis of root system was not performed.

## Conclusions

The lowland ecotype exhibited significant reductions in terminal shoot growth and higher seedling mortality under drought treatment in contrast to the upland ecotype. Needle water content, LMA, and chlorophyll content differed between ecotypes, with the upland ecotype generally maintaining higher values and better photosynthetic resilience. Drought reduced needle cross-section area and triggered structural adaptations in both ecotypes, though more strongly in the lowland ecotype, again pointing to the better drought resilience of the upland ecotype. Chlorophyll fluorescence parameters also revealed that the upland ecotype recovered more efficiently and proved greater resilience under early season drought stress. We conclude that the upland ecotype showed several functional traits corresponding to better resilience to drought stress and recovery as compared to the lowland ecotype.

Understanding the response of plant functional traits to environmental variability is essential for early-age nursery seedling screening [117], which can enhance supporting reforestation efforts to combat drought and ensure survival of European forests in conditions of climate change [118]. Thus, developing fast, non-destructive, effective, and automated methods, especially optical detection, for identifying functional leaf traits with the aim of distinguishing more resistant or resilient ecotypes already in their seedling phase is currently a key focus for plant biologists, forest researchers, ecologists, and geneticists. Such findings then serve for nursery practice and employment of high-throughput phenotyping unit [33]. Furthermore, recent research highlights the critical importance of maintaining high inter- and intra-population genetic variability, as this provides the adaptive potential necessary to respond to fluctuating environmental conditions [119] and at the same time harbours future suitable resources for assisted migration into target environments.

## Supplementary Information

Supplementary Material 1.

## Data Availability

The datasets analysed during the current study are available from the corresponding author on reasonable request.
