# Supplementary material for: Scots pine seedlings of lowland and upland ecotypes respond differently to drought detected by needle functional traits and spectral reflectance
Source: BMC Plant Biol. 2026 Jan 14;26:261. doi: 10.1186/s12870-025-08019-y (PMC12888561; doi:10.1186/s12870-025-08019-y)
Supplement: Supplementary file 1 — Supplementary Material 1. [file 12870_2025_8019_MOESM1_ESM.docx]

**Scots pine seedlings of lowland and upland ecotypes respond differently to drought detected by needle functional traits and spectral reflectance**

Kristýna Štěpánová^1^, Zuzana Lhotáková^1^, Eva Neuwirthová^1^, Lucie Kupková^3^, Lucie Červená^3^, Filip Raasch^3^, Markéta Potůčková^3^, Jan Stejskal^2^, Jaroslav Čepl^2^, Petya Campbell ^4^, Milan Lstibůrek^2^, Jana Albrechtová^1^**

^1^  Department of Experimental Plant Biology, Faculty of Science, Charles University, Viničná 5, 12844 Prague, Czech Republic

^2^ Department of Forest Genetics and Physiology, Faculty of Forestry and Wood Sciences, Czech University of Life Sciences Prague, Prague, Czech Republic

^3^ Department of Applied Geoinformatics and Cartography, Faculty of Science, Charles University, Albertov 6, 12800 Prague, Czech Republic

^4^ University of Maryland Baltimore County and Department of Geography and Environmental Sciences and NASA/Goddard Space Flight Center, Biospheric Sciences Laboratory, Greenbelt, MD 20771 USA

****** Correspondence: jana.albrechtova@natur.cuni.cz

# Supporting information

Additional Supporting Information may be found online in the Supporting Information section at the end of the article.

## Figure S1 Schematic visualization of the fluorescence induction quenching protocol

**
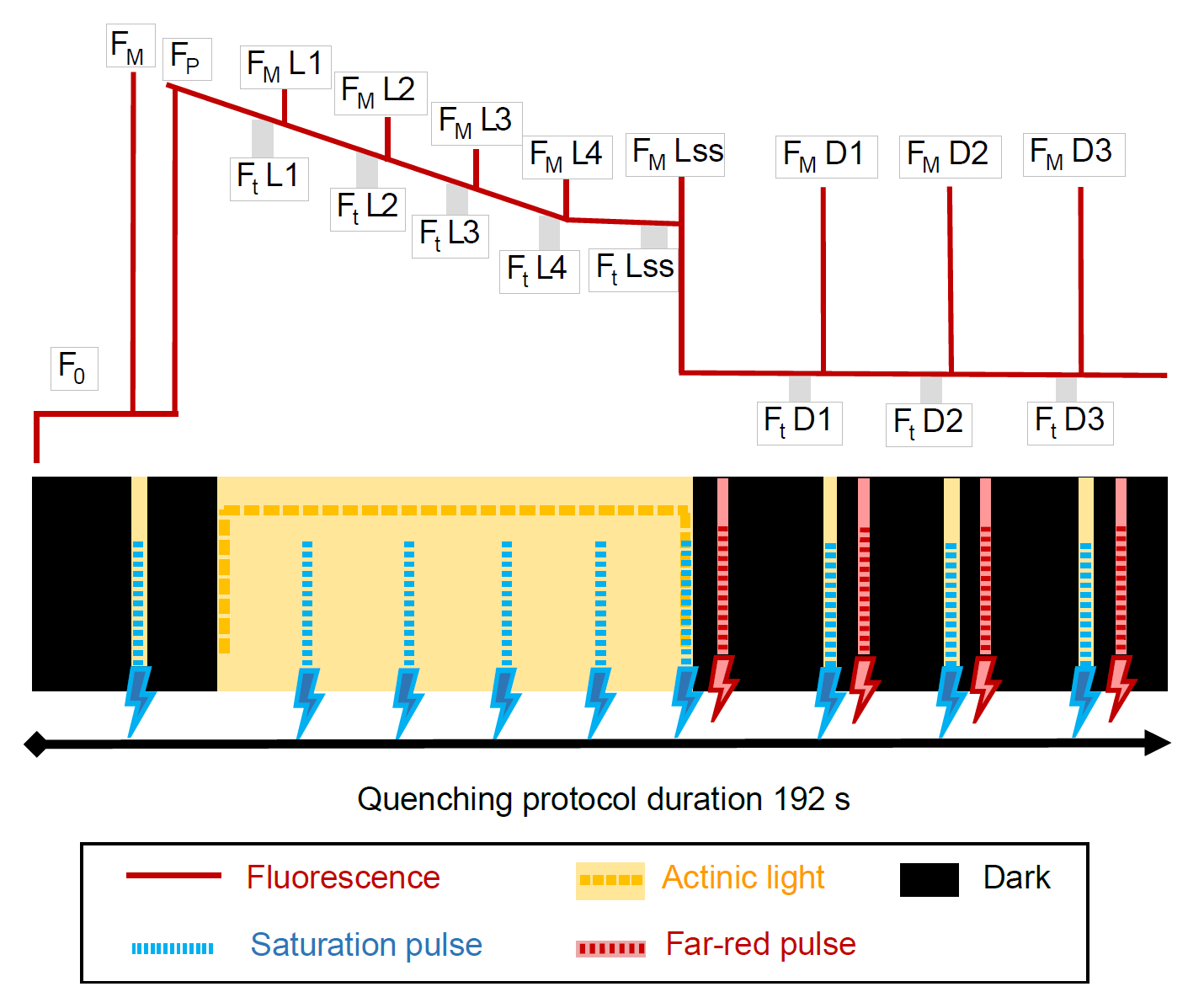
**

**Figure S1** Schematic visualization of the fluorescence induction quenching protocol. The red line in the upper part represents chlorophyll fluorescence intensity during the 192 s duration of the pulse-amplitude-modulated mode. The lower part of the diagram corresponds to the approximate timing of the periods of the dark (in black), continuous actinic light (yellow), saturation flashes (dashed blue) and far-red pulses (dashed red). The measured fluorescence quantities are indicated in grey frames: F_0_ – minimal fluorescence of a dark-adapted plant, F_M_ – maximal fluorescence after exposition to the saturation flash in dark, F_M_ L1-4 - during light adaptation, F_M_ Lss – in light-adapted steady state, and F_M_ D1-3 during dark adaptation. F_P_ - peak fluorescence during the first seconds of the transient when the plastoquinone pool is mostly reduced and neither the photoprotective mechanisms, nor the CO_2_ assimilation, are fully activated, F_t_ L1-4 – fluorescence intensity during light adaptation, F_t_ Lss – fluorescence intensity in the light-adapted steady state, F_M_ D1-3 fluorescence intensity during dark adaptation; adapted from (Photon Systems Instruments, 2019).

## Figure S2 Scheme of the measurement setup for spectral reflectance acquisition

**
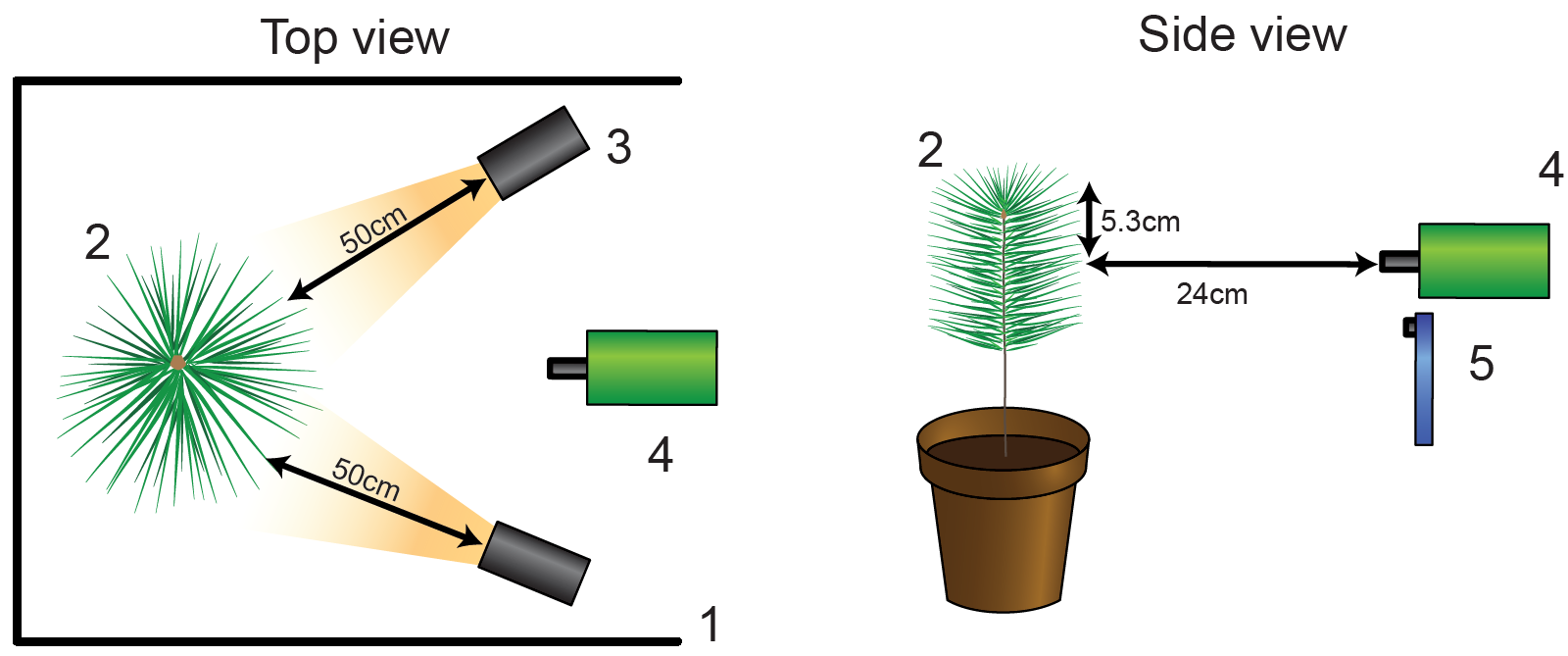
**

**Figure S2** Custom made measurement setup for spectral reflectance acquisition from Scots pine seedlings – Top and Side views. 1 – spectrally black box with one side open, 2 – Scots pine seedling, 3 - halogen lamp, 4 - pistol grip holding optical cable, 5 - cell phone for taking photos of field of view of the optical cable.

## Table S1 Effect of drought on the terminal shoot length in the current year.

|  | **p-value** | | | | | |
| --- | --- | --- | --- | --- | --- | --- |
| **Factor** |  | Stress | |  | | Recovery |
| Treatment | | | 0.093 | | 0.005 | |
| Ecotype | | | 0.364 | | 0.668 | |
| DAT | | | <0.001 | | <0.001 | |
| Treatment:Ecotype | | | 0.551 | | <0.001 | |
| Treatment:DAT | | | 0.236 | | 0.808 | |
| Ecotype:DAT | | | 0.408 | | 0.795 | |
| Treatment:Ecotype:DAT | | | 0.008 | | 0.972 | |

**Table S1** Effect of ecotype, drought treatment, time, and their interactions on the length of terminal shoot in stress and recovery period. Significant p-values (< 0.05) are marked in bold. n = 16-17 per each combination of ecotype and treatment.

## Tables S2 and S3 Basic statistical characteristics of measured parameters

|  |  |  | Stres NAC2 (DAT 43 - 78) | | | | Recovery NAC2 (DAT 99) | | | | Recovery NAC1 (DAT 161) | | | |
| --- | --- | --- | --- | --- | --- | --- | --- | --- | --- | --- | --- | --- | --- | --- |
| Parameter | Ecotype | Treatment | **Min** | **Max** | **Median** | **Mean** | **Min** | **Max** | **Median** | **Mean** | **Min** | **Max** | **Median** | **Mean** |
| LMA | L | C | 0.016 | 0.025 | 0.019 | 0.020 | 0.043 | 0.060 | 0.050 | 0.051 | 0.011 | 0.016 | 0.014 | 0.014 |
|  |  | MD | 0.017 | 0.024 | 0.020 | 0.020 | 0.033 | 0.059 | 0.044 | 0.044 | 0.010 | 0.017 | 0.012 | 0.013 |
|  |  | D | 0.016 | 0.026 | 0.022 | 0.022 | 0.040 | 0.060 | 0.051 | 0.050 | 0.008 | 0.015 | 0.010 | 0.011 |
|  | U | C | 0.015 | 0.024 | 0.019 | 0.020 | 0.039 | 0.054 | 0.042 | 0.044 | 0.007 | 0.015 | 0.011 | 0.011 |
|  |  | MD | 0.015 | 0.025 | 0.021 | 0.020 | 0.038 | 0.055 | 0.045 | 0.046 | 0.008 | 0.017 | 0.011 | 0.011 |
|  |  | D | 0.017 | 0.022 | 0.022 | 0.022 | 0.036 | 0.054 | 0.049 | 0.047 | 0.006 | 0.014 | 0.010 | 0.010 |
| Water | L | C | 0.573 | 0.658 | 0.638 | 0.633 | 0.595 | 0.685 | 0.645 | 0.644 | 0.565 | 0.736 | 0.662 | 0.665 |
|  |  | MD | 0.592 | 0.653 | 0.628 | 0.628 | 0.615 | 0.728 | 0.664 | 0.671 | 0.583 | 0.730 | 0.696 | 0.673 |
|  |  | D | 0.455 | 0.638 | 0.593 | 0.589 | 0.608 | 0.696 | 0.635 | 0.644 | 0.690 | 0.790 | 0.727 | 0.730 |
|  | U | C | 0.625 | 0.660 | 0.637 | 0.641 | 0.611 | 0.727 | 0.671 | 0.665 | 0.657 | 0.798 | 0.698 | 0.709 |
|  |  | MD | 0.586 | 0.665 | 0.631 | 0.631 | 0.621 | 0.716 | 0.673 | 0.669 | 0.659 | 0.785 | 0.707 | 0.713 |
|  |  | D | 0.538 | 0.641 | 0.608 | 0.606 | 0.621 | 0.725 | 0.653 | 0.665 | 0.670 | 0.821 | 0.744 | 0.742 |
| Chl a+b | L | C | 3.500 | 6.080 | 5.000 | 4.950 | 3.970 | 6.140 | 4.690 | 4.830 | 0.100 | 9.090 | 5.560 | 5.730 |
|  |  | MD | 4.090 | 6.330 | 5.060 | 5.180 | 4.320 | 7.430 | 5.420 | 5.700 | 5.130 | 13.800 | 7.800 | 7.920 |
|  |  | D | 3.000 | 4.950 | 4.570 | 4.260 | 4.120 | 5.800 | 5.090 | 5.060 | 6.780 | 13.700 | 10.200 | 9.800 |
|  | U | C | 5.040 | 6.460 | 5.620 | 5.640 | 5.070 | 7.950 | 6.160 | 6.260 | 5.280 | 15.100 | 8.670 | 9.490 |
|  |  | MD | 4.870 | 7.270 | 6.050 | 6.080 | 4.680 | 8.470 | 6.930 | 6.590 | 4.330 | 13.600 | 7.550 | 8.410 |
|  |  | D | 4.200 | 6.560 | 5.250 | 5.210 | 4.790 | 7.580 | 5.800 | 6.250 | 6.650 | 16.900 | 10.600 | 11.400 |
| Car | L | C | 0.418 | 0.718 | 0.618 | 0.609 | 0.506 | 0.854 | 0.647 | 0.653 | 0.013 | 1.150 | 0.750 | 0.743 |
|  |  | MD | 0.546 | 0.736 | 0.617 | 0.624 | 0.585 | 0.945 | 0.731 | 0.754 | 0.654 | 1.900 | 0.931 | 1.020 |
|  |  | D | 0.452 | 0.646 | 0.565 | 0.561 | 0.545 | 0.779 | 0.704 | 0.688 | 0.876 | 2.500 | 1.320 | 1.420 |
|  | U | C | 0.594 | 0.865 | 0.728 | 0.725 | 0.660 | 1.080 | 0.834 | 0.841 | 0.694 | 2.140 | 1.110 | 1.240 |
|  |  | MD | 0.637 | 0.889 | 0.756 | 0.756 | 0.624 | 1.150 | 0.929 | 0.884 | 0.546 | 1.860 | 0.942 | 1.070 |
|  |  | D | 0.569 | 0.861 | 0.680 | 0.680 | 0.683 | 1.030 | 0.811 | 0.851 | 0.860 | 2.420 | 1.530 | 1.620 |
| Chla/b | L | C | 2.270 | 2.540 | 2.460 | 2.440 | 2.280 | 2.550 | 2.480 | 2.460 | 2.260 | 2.600 | 2.440 | 2.440 |
|  |  | MD | 2.260 | 2.620 | 2.380 | 2.400 | 2.270 | 2.550 | 2.410 | 2.420 | 2.280 | 2.650 | 2.430 | 2.450 |
|  |  | D | 2.160 | 2.490 | 2.370 | 2.350 | 2.140 | 2.450 | 2.360 | 2.340 | 2.490 | 2.790 | 2.550 | 2.580 |
|  | U | C | 2.300 | 2.510 | 2.410 | 2.400 | 2.260 | 2.570 | 2.450 | 2.440 | 2.320 | 2.670 | 2.440 | 2.460 |
|  |  | MD | 2.250 | 2.500 | 2.410 | 2.390 | 2.230 | 2.570 | 2.390 | 2.410 | 2.270 | 2.680 | 2.470 | 2.460 |
|  |  | D | 2.200 | 2.390 | 2.350 | 2.330 | 2.150 | 2.530 | 2.390 | 2.370 | 2.440 | 2.680 | 2.520 | 2.550 |
| Car/Chla+b | L | C | 0.117 | 0.136 | 0.122 | 0.123 | 0.127 | 0.144 | 0.134 | 0.135 | 0.120 | 0.139 | 0.132 | 0.132 |
|  |  | MD | 0.111 | 0.134 | 0.119 | 0.121 | 0.122 | 0.140 | 0.134 | 0.133 | 0.115 | 0.142 | 0.126 | 0.128 |
|  |  | D | 0.123 | 0.144 | 0.133 | 0.133 | 0.129 | 0.147 | 0.137 | 0.137 | 0.119 | 0.138 | 0.130 | 0.130 |
|  | U | C | 0.114 | 0.133 | 0.124 | 0.124 | 0.129 | 0.144 | 0.135 | 0.134 | 0.121 | 0.142 | 0.130 | 0.130 |
|  |  | MD | 0.116 | 0.134 | 0.123 | 0.124 | 0.128 | 0.139 | 0.134 | 0.134 | 0.112 | 0.140 | 0.128 | 0.128 |
|  |  | D | 0.119 | 0.145 | 0.131 | 0.131 | 0.131 | 0.143 | 0.137 | 0.137 | 0.120 | 0.142 | 0.134 | 0.133 |

**Table S2** Summary statistics of measured values of selected functional traits (Water, LMA, Chl a+b, Car, Chla/Chlb, Car/Chl a+b) of previous-year (NAC2) and current-year needles (NAC1). Sampling of NAC2 needles was done at the end of the stress period (DAT 78) and after two weeks of rewatering (DAT 99), sampling of NAC1 needles was done at the end of recovery period (DAT 161). n = 24 - 28 per each combination of ecotype and treatment for NAC2 needles and n = 8 - 15 per each combination of ecotype (Lowland – L; Upland – U) and treatment (Control – C; Mild Drought – MD; Drought – D) for NAC1 needles.

|  |  |  | Stres NAC2 (DAT 43 - 78) | | | | Recovery NAC2 (DAT 84 + 99) | | | | Recovery NAC1 (DAT 120 + 145) | | | |
| --- | --- | --- | --- | --- | --- | --- | --- | --- | --- | --- | --- | --- | --- | --- |
| Parameter | Ecotype | Treatment | **Min** | **Max** | **Median** | **Mean** | **Min** | **Max** | **Median** | **Mean** | **Min** | **Max** | **Median** | **Mean** |
| QY_max_ | L | C | 0.780 | 0.850 | 0.820 | 0.822 | 0.815 | 0.835 | 0.827 | 0.825 | 0.810 | 0.830 | 0.830 | 0.824 |
|  |  | MD | 0.800 | 0.850 | 0.820 | 0.824 | 0.810 | 0.830 | 0.820 | 0.820 | 0.820 | 0.840 | 0.830 | 0.830 |
|  |  | D | 0.800 | 0.850 | 0.820 | 0.821 | 0.810 | 0.830 | 0.815 | 0.818 | 0.810 | 0.840 | 0.835 | 0.831 |
|  | U | C | 0.790 | 0.840 | 0.820 | 0.819 | 0.800 | 0.835 | 0.820 | 0.821 | 0.810 | 0.830 | 0.825 | 0.823 |
|  |  | MD | 0.780 | 0.840 | 0.820 | 0.819 | 0.810 | 0.835 | 0.822 | 0.823 | 0.810 | 0.840 | 0.830 | 0.827 |
|  |  | D | 0.780 | 0.830 | 0.817 | 0.815 | 0.815 | 0.825 | 0.818 | 0.819 | 0.810 | 0.830 | 0.830 | 0.825 |
| F_V_F_M_ Lss | L | C | 0.590 | 0.760 | 0.680 | 0.681 | 0.630 | 0.725 | 0.680 | 0.680 | 0.560 | 0.610 | 0.570 | 0.579 |
|  |  | MD | 0.600 | 0.740 | 0.655 | 0.659 | 0.610 | 0.690 | 0.660 | 0.655 | 0.570 | 0.660 | 0.575 | 0.597 |
|  |  | D | 0.590 | 0.740 | 0.670 | 0.667 | 0.590 | 0.690 | 0.655 | 0.656 | 0.570 | 0.650 | 0.605 | 0.604 |
|  | U | C | 0.600 | 0.740 | 0.660 | 0.668 | 0.590 | 0.670 | 0.633 | 0.631 | 0.570 | 0.640 | 0.570 | 0.586 |
|  |  | MD | 0.580 | 0.730 | 0.675 | 0.664 | 0.610 | 0.685 | 0.633 | 0.644 | 0.570 | 0.660 | 0.590 | 0.596 |
|  |  | D | 0.565 | 0.750 | 0.650 | 0.651 | 0.605 | 0.675 | 0.647 | 0.643 | 0.570 | 0.640 | 0.615 | 0.610 |
| QYL1 | L | C | 0.220 | 0.330 | 0.285 | 0.284 | 0.260 | 0.315 | 0.295 | 0.291 | 0.200 | 0.280 | 0.240 | 0.233 |
|  |  | MD | 0.210 | 0.320 | 0.270 | 0.271 | 0.245 | 0.305 | 0.278 | 0.274 | 0.200 | 0.280 | 0.220 | 0.226 |
|  |  | D | 0.240 | 0.320 | 0.280 | 0.282 | 0.240 | 0.300 | 0.265 | 0.268 | 0.200 | 0.270 | 0.240 | 0.235 |
|  | U | C | 0.230 | 0.310 | 0.265 | 0.270 | 0.235 | 0.325 | 0.260 | 0.269 | 0.200 | 0.270 | 0.250 | 0.236 |
|  |  | MD | 0.220 | 0.320 | 0.260 | 0.263 | 0.210 | 0.270 | 0.250 | 0.246 | 0.200 | 0.250 | 0.210 | 0.217 |
|  |  | D | 0.200 | 0.320 | 0.252 | 0.249 | 0.210 | 0.260 | 0.236 | 0.235 | 0.200 | 0.280 | 0.245 | 0.243 |
| QY Lss | L | C | 0.130 | 0.290 | 0.210 | 0.209 | 0.155 | 0.230 | 0.198 | 0.193 | 0.100 | 0.190 | 0.120 | 0.131 |
|  |  | MD | 0.120 | 0.270 | 0.170 | 0.180 | 0.150 | 0.235 | 0.190 | 0.191 | 0.100 | 0.200 | 0.150 | 0.134 |
|  |  | D | 0.120 | 0.250 | 0.180 | 0.187 | 0.120 | 0.240 | 0.190 | 0.189 | 0.100 | 0.210 | 0.150 | 0.149 |
|  | U | C | 0.150 | 0.270 | 0.200 | 0.204 | 0.145 | 0.235 | 0.200 | 0.195 | 0.100 | 0.180 | 0.120 | 0.129 |
|  |  | MD | 0.110 | 0.270 | 0.210 | 0.203 | 0.155 | 0.230 | 0.205 | 0.196 | 0.100 | 0.200 | 0.130 | 0.138 |
|  |  | D | 0.070 | 0.280 | 0.190 | 0.189 | 0.125 | 0.205 | 0.172 | 0.169 | 0.100 | 0.190 | 0.190 | 0.168 |
| NPQ Lss | L | C | 0.610 | 2.210 | 1.210 | 1.260 | 0.960 | 1.960 | 1.280 | 1.380 | 2.250 | 3.050 | 2.610 | 2.640 |
|  |  | MD | 0.740 | 2.120 | 1.630 | 1.560 | 1.240 | 2.170 | 1.620 | 1.650 | 1.650 | 3.150 | 2.400 | 2.420 |
|  |  | D | 0.810 | 1.980 | 1.350 | 1.420 | 0.720 | 1.950 | 1.480 | 1.440 | 1.990 | 2.780 | 2.260 | 2.290 |
|  | U | C | 0.730 | 2.050 | 1.400 | 1.360 | 1.240 | 2.160 | 1.520 | 1.660 | 2.140 | 2.970 | 2.660 | 2.580 |
|  |  | MD | 0.880 | 2.290 | 1.380 | 1.350 | 1.220 | 2.260 | 1.810 | 1.740 | 1.910 | 2.770 | 2.140 | 2.280 |
|  |  | D | 0.680 | 1.900 | 1.510 | 1.430 | 1.500 | 2.240 | 1.670 | 1.770 | 1.700 | 2.860 | 2.160 | 2.220 |
| Rfd Lss | L | C | 0.460 | 1.620 | 0.910 | 0.916 | 0.670 | 1.360 | 0.950 | 1.000 | 1.590 | 1.900 | 1.740 | 1.720 |
|  |  | MD | 0.520 | 1.700 | 1.080 | 1.090 | 0.860 | 1.460 | 1.130 | 1.120 | 1.230 | 1.770 | 1.480 | 1.510 |
|  |  | D | 0.600 | 1.380 | 1.020 | 0.973 | 0.660 | 1.320 | 0.960 | 0.998 | 1.420 | 1.850 | 1.570 | 1.580 |
|  | U | C | 0.650 | 1.480 | 1.020 | 1.020 | 0.930 | 1.460 | 1.200 | 1.180 | 1.620 | 2.050 | 1.820 | 1.830 |
|  |  | MD | 0.700 | 1.530 | 1.040 | 1.050 | 0.950 | 1.600 | 1.280 | 1.300 | 1.340 | 1.920 | 1.540 | 1.560 |
|  |  | D | 0.580 | 2.380 | 1.160 | 1.180 | 1.130 | 1.560 | 1.270 | 1.310 | 1.430 | 1.880 | 1.490 | 1.570 |

**Table S3** Summary statistics of measured values of selected chlorophyll fluorescence parameters calculated from the quenching protocol in stress and recovery period. QY_max_ – the maximum quantum fluorescence yield of dark-adapted needles, F_V_/F_M_ LSS - PSII maximum efficiency of light adapted sample in steady-state, QY L1 – PSII quantum yield induced in light outside the saturation pulse, QY Lss – PSII quantum yield in steady-state in light outside the saturation pulse, NPQ Lss - Steady-state non-photochemical quenching, Rfd Lss – fluorescence drop ratio on steady state. Six measurements were taken on previous year-needles (NAC2) during the stress period and two measurements at the beginning of the recovery period; two measurements were taken on the current-needles (NAC1) during the recovery period. n = 3-6 per each combination of ecotype (Lowland – L; Upland – U) and treatment (Control – C; Mild Drought – MD; Drought – D).

## Figures S3 and S4 Vegetation indices

**
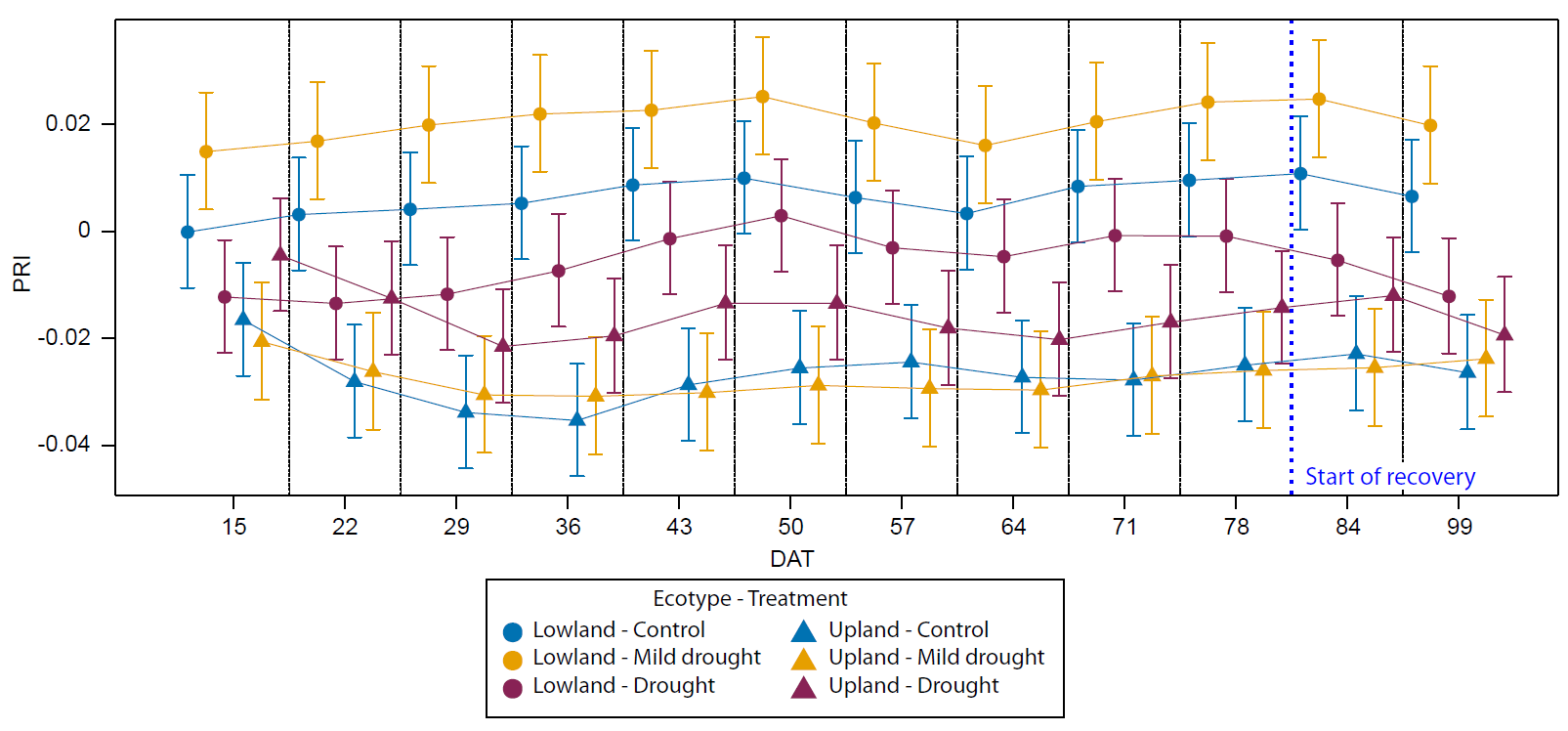
**

**Figure S3** The photochemical reflectance index (PRI). X-axis: days after transplantation (DAT 15-99). Weakly measurements during the stress and recovery period until the DAT 84, later in the recovery period the measurement interval was longer. The vertical red dashed line marks the beginning of the recovery period at 24^th^ June (DAT 78). Symbols represent the average predicted values for the PRI of NAC2 needles of upland and lowland Scots pine ecotypes with different water treatments (different colours), obtained by univariate linear mixed model with predicted 95% confidence interval. n = 25-30 per each combination of ecotype and treatment.


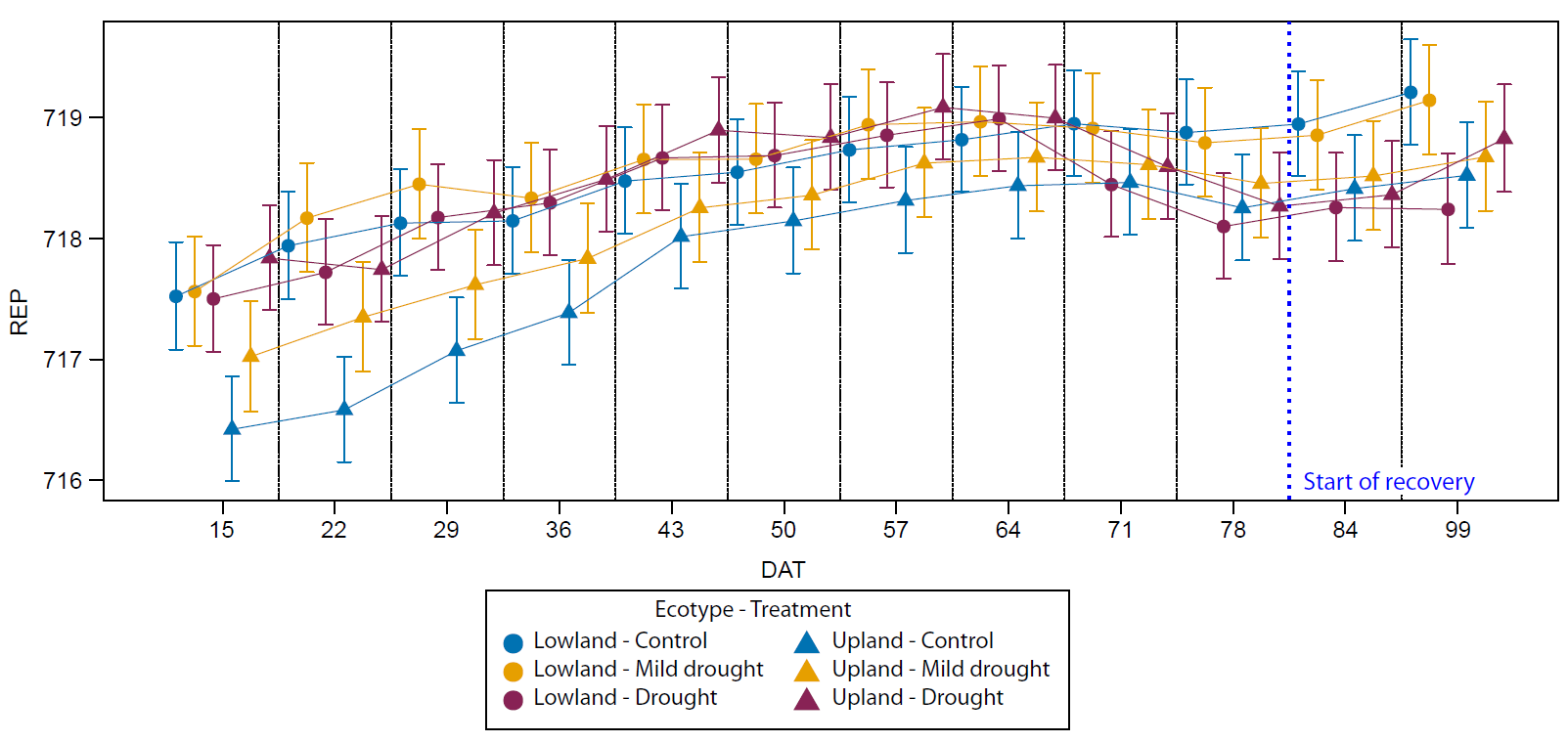


**Figure S4** The red edge position (REP). X-axis: days after transplantation (DAT 15-99). Weakly measurements during the stress and recovery period until the DAT 84, later in the recovery period the measurement interval was longer. The vertical red dashed line marks the beginning of the recovery period at 24^th^ June (DAT 78). Symbols represent the average predicted values for the REP of NAC2 needles of upland and lowland Scots pine ecotypes with different water treatments (different colours), obtained by univariate linear mixed model with predicted 95% confidence interval. n = 25-30 per each combination of ecotype and treatment.

## Figures S5, S6 and S7 Cohen’s d and adjusted p-values for pairwise treatment comparisons


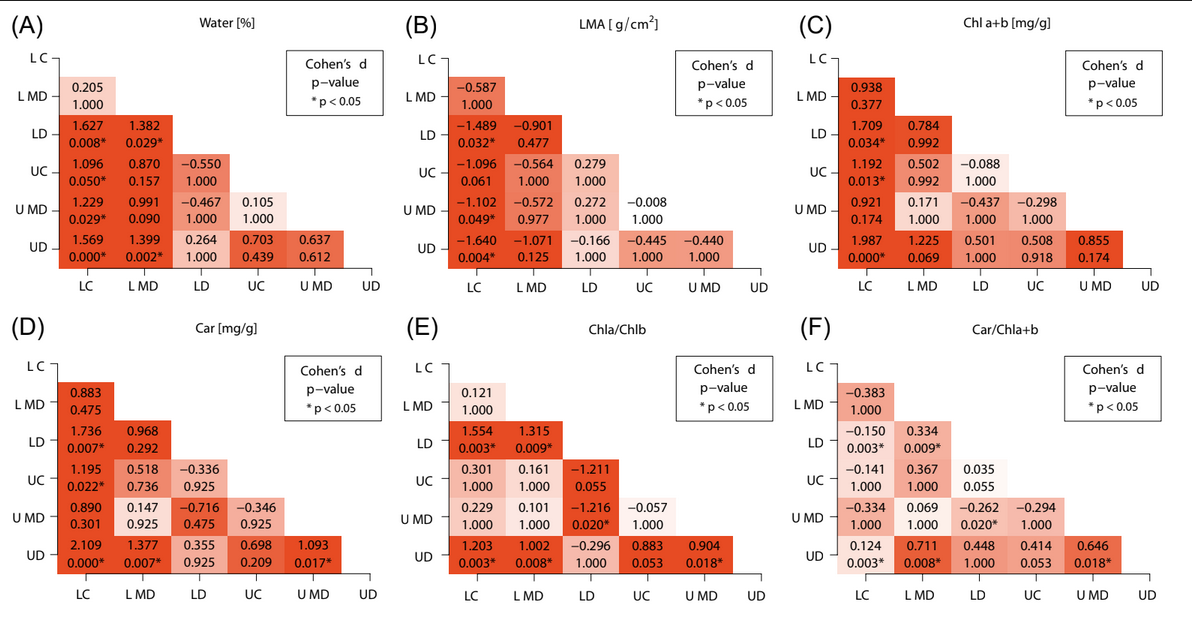


**Figure S5** Cohen’s d (top) and p-values (bottom) of selected functional traits of current-year needles (NAC1) at the end of the growing season (DAT 161). Water content (**A**), leaf mass per area (**B**), content of chlorophyll *a* + *b* (**C**) and carotenoids (**D**), ratio of chlorophyll *a* and chlorophyll *b* (**E**), ratio of carotenoids and chlorophyll *a* + *b* (**F**). Comparisons across Treatment × Ecotype combinations. Axes represent factor combinations: of ecotype (lowland – L and upland – U) and treatment (Control – C; Mild drought – MD; Drought – D). Stars indicate statistically significant differences (α = 0.05). Color intensity reflects the absolute value of *Cohen’s d* (darker = larger effect); numbers indicate direction – positive = higher mean in the row group, negative = higher mean in the column group.


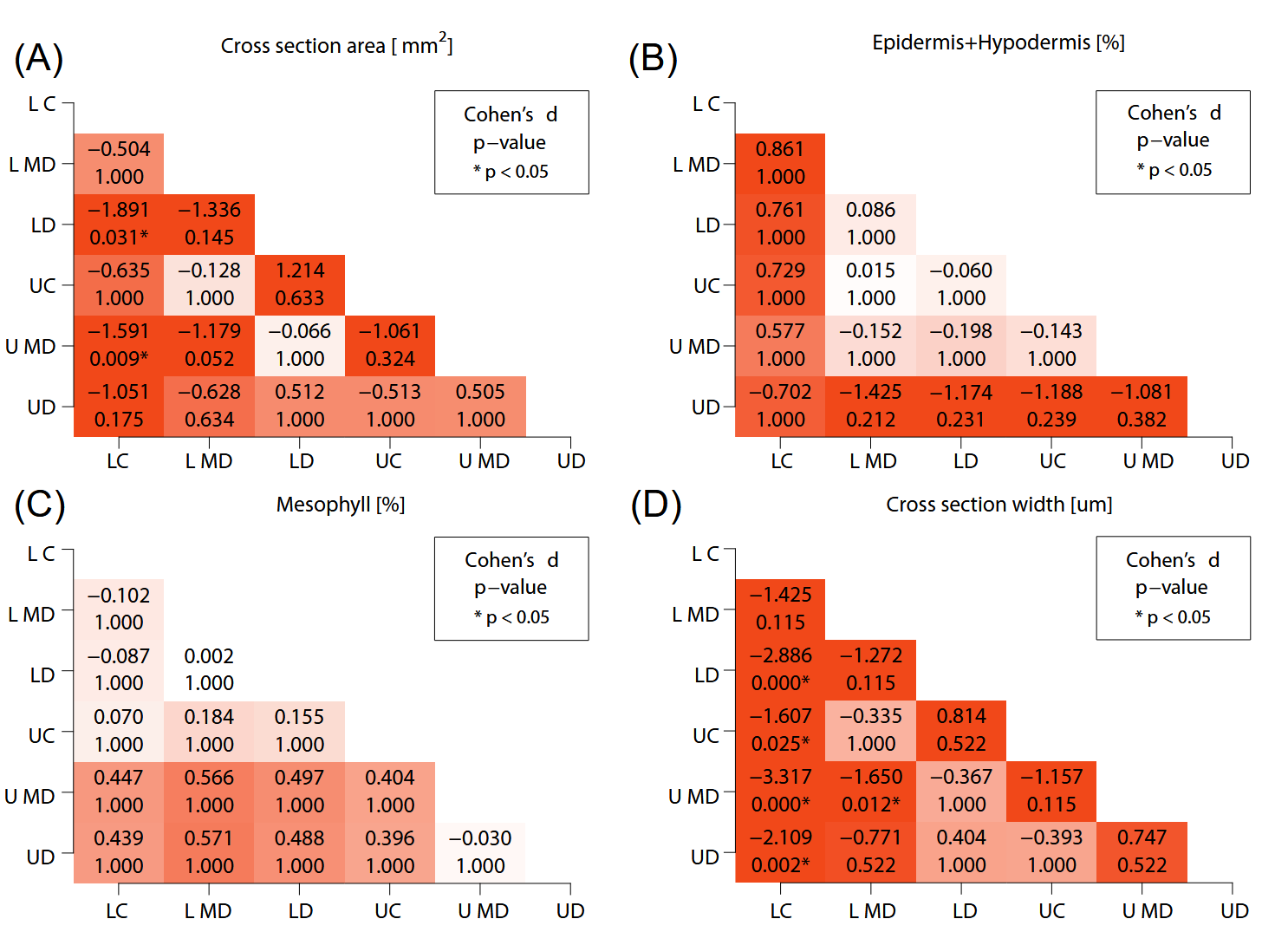


**Figure S6** Cohen’s d (top) and p-values (bottom) of selected needle anatomical traits evaluated on the current-year needles (NAC1) at the end of the growing season (DAT 161). Cross-section area (**A**), Epidermis and hypodermis proportion on needle cross-section area in percentage (**B**), mesophyll proportion on needle cross-section area in percentage (**C**), cross-section width (**D**). Comparisons across Treatment × Ecotype combinations. Axes represent factor combinations: of ecotype (lowland – L and upland – U) and treatment (Control – C; Mild drought – MD; Drought – D). Stars indicate statistically significant differences (α = 0.05). Color intensity reflects the absolute value of *Cohen’s d* (darker = larger effect); numbers indicate direction – positive = higher mean in the row group, negative = higher mean in the column group.

**
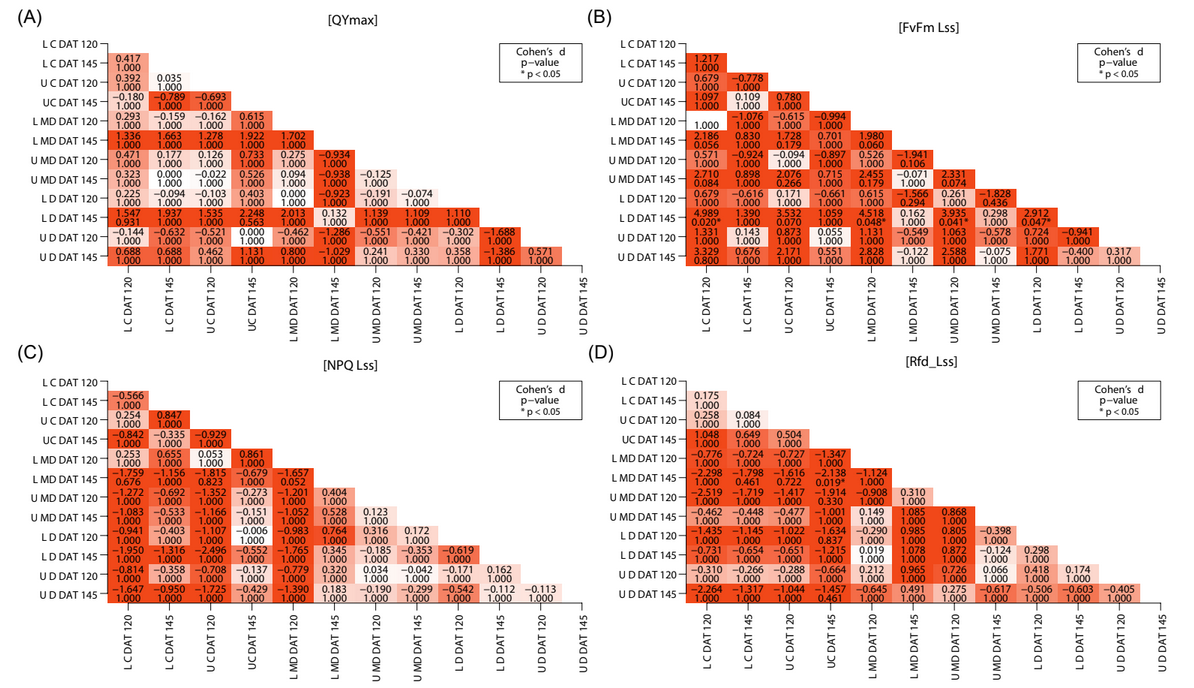
**

**Figure S7** Heatmap of Cohen’s d (top) and p-values (bottom) of selected chlorophyll fluorescence parameters evaluated on the current-year needles (NAC1) calculated from the Quenching protocol – QY_max_ (**A**), F_V_/F_M_ L_SS_ (**B**), NPQ L_SS_ (**C**), Rfd L_SS_ (**D**). Comparisons across Treatment × Ecotype × DAT combinations. Axes represent factor combinations: of ecotype (lowland – L and upland – U), treatment (Control – C; Mild drought – MD; Drought – D), and measurement date (DAT 120 and 145). Stars indicate statistically significant differences (α = 0.05). Color intensity reflects the absolute value of *Cohen’s d* (darker = larger effect); numbers indicate direction – positive = higher mean in the row group, negative = higher mean in the column group.

##
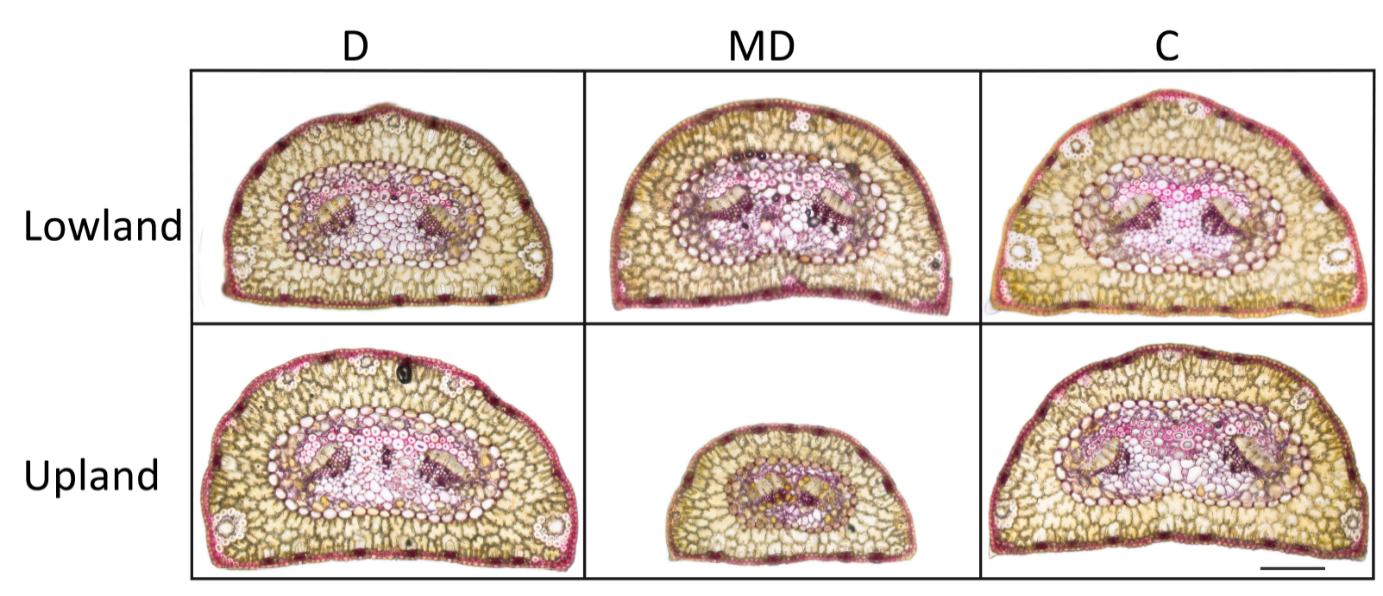
Figure S8 Needle anatomy

**Figure S8** Scots pine needle cross-section. Selected images with representatives from each combination of two ecotypes (lowland and upland) and treatment (Control – C; Mild drought – MD; Drought – D). Needle arched abaxial side is oriented in upper direction, needle adaxial side is oriented in down direction, Cuticle, epidermis, and lignified hypodermis on the needle surface are stained red. Arm-shaped palisade cells of mesophyll are below without staining, central cylinder comprises vascular bundle), round-shaped resin ducts are located below the covering tissues, usually in a corner of junction of adaxial and abaxial epidermis.. Free-hand section, phloroglucinol - HCl staining (lignified cell walls stain cherry red). The bar corresponds to 100 µm.
